# Traditional risk factors and premature acute coronary syndromes in South Eastern Europe: a multinational cohort study

**DOI:** 10.1016/j.lanepe.2023.100824

**Published:** 2024-01-02

**Authors:** Raffaele Bugiardini, Edina Cenko, Jinsung Yoon, Maria Bergami, Zorana Vasiljevic, Guiomar Mendieta, Marija Zdravkovic, Marija Vavlukis, Sasko Kedev, Davor Miličić, Lina Badimon, Olivia Manfrini

**Affiliations:** a Department of Medical and Surgical Sciences, University of Bologna, Bologna, Italy; b Google Cloud AI, Sunnyvale, California, USA; c Medical Faculty, University of Belgrade, Belgrade, Serbia; d Servei de Cardiologia, Institut Clínic Cardiovascular, Hospital Clínic de Barcelona, Barcelona, Spain & Institut d’Investigacions Biomèdiques August Pi i Sunyer (IDIBAPS), Barcelona, Spain; e Faculty of Medicine, Clinical Hospital Center Bezanijska Kosa, University of Belgrade, Belgrade, Serbia; f University Clinic for Cardiology, Skopje 1000, Republic of North Macedonia; g Faculty of Medicine, Ss. Cyril and Methodius University in Skopje, Skopje 1000, Republic of North Macedonia; h Department for Cardiovascular Diseases, University Hospital Center Zagreb, University of Zagreb, Zagreb, Croatia; i Cardiovascular Research Program ICCC, IR-IIB Sant Pau, Hospital de la Santa Creu i Sant Pau, CiberCV-Institute Carlos III, Barcelona, Spain; j IRCCS Azienda Ospedaliero-Universitaria di Bologna Sant’Orsola Hospital, Bologna, Italy

**Keywords:** Risk factors, Sex differences, Ischemic heart disease

## Abstract

**Background:**

The age-standardized death rates under 65 years from ischemic heart disease in South Eastern Europe are approximately twice as high than the Western Europe average, but the reasons are not completely recognized. The aim of the present study was to address this issue by collecting and analyzing data from a large, multinational cohort.

**Methods:**

We enrolled 70,953 Caucasian patients with first acute coronary syndrome, from 36 urban hospital in 7 South Eastern European countries and assessed their life expectancy free of acute coronary syndrome and mortality within 30 days after hospital admission from acute coronary syndrome as estimated in relation to dichotomous categories of traditional risk factors (current smoking, hypertension, diabetes and hypercholesterolemia) stratified according to sex.

**Findings:**

Compared with patients without any baseline traditional risk factors, the presence of all four risk factors was associated with a 5-year shorter life expectancy free of acute coronary syndrome (women: from 67.1 ± 12.0 to 61.9 ± 10.3 years; *r* = −0.089; p < 0.001 and men: from 62.8 ± 12.2 to 58.9 ± 9.9 years; *r* = −0.096; p < 0.001). Premature acute coronary syndrome (women <67 years and men <63 years) was remarkably related to current smoking and hypercholesterolemia among women (RRs: 3.96; 95% CI: 3.72–4.20 and 1.31; 95% CI: 1.25–1.38, respectively) and men (RRs: 2.82; 95% CI: 2.71–2.93 and 1.39; 95% CI: 1.34–1.45, respectively). Diabetes was most strongly associated with death from premature acute coronary syndrome either in women (RR: 1.52; 95% CI: 1.29–1.79) or men (RR: 1.63; 95% CI: 1.41–1.89).

**Interpretation:**

Public health policies in South Eastern Europe should place significant emphasis on the four traditional risk factors and the associated lifestyle behaviors to reduce the epidemic of premature ischemic heart disease.

**Funding:**

None.

**Research in context**

**Evidence before this study**

Following a literature search conducted up to October 1st 2023, we identified published papers in English language from academic databases, mainly MEDLINE, through PubMed search using keywords such as “South Eastern Europe”, “Balkans”, “Eastern Europe”, “former Soviet Union”, “IHD mortality”, “premature death” and their combinations. We also searched for any relevant mortality data from the World Health Organization (WHO) and the European Cardiovascular Disease Statistics. There are few registry data from South Eastern European countries. The European Cardiovascular Disease Statistics and the WHO European Mortality Database assess the impact of ischemic heart disease on premature deaths by calculating the proportion of age-standardized mortality rate under 65 years per 100,000 inhabitants. The age-standardized death rates under 65 years are approximately twice as high in South Eastern Europe than the Western Europe average. Acute coronary syndrome is the main driver of premature mortality in ischemic heart disease. As such, acute coronary syndrome is a significant public health concern in South Eastern Europe. Previous studies relating traditional risk factors (current smoking, diabetes, hypercholesterolemia and hypertension) with acute coronary syndrome and mortality have been mainly restricted to populations from North America and Western Europe. There is a lack of information on the relationship between traditional risk factors and premature acute coronary syndrome and death from premature acute coronary syndrome in South Eastern Europe. This gap in knowledge underscores a critical need for large studies with individual data and standardized methodologies involving multiple South Eastern European countries, which may provide valuable insights into the impact of traditional risk factors on disease premature incidence and mortality.

**Added value of this study**

We harmonized individual-level data and compared the associations of the four traditional risk factors with premature acute coronary syndrome and mortality from premature acute coronary syndrome in 70,953 Caucasian patients who did not have a prior history of cardiovascular disease enrolled from 36 urban hospital in 7 South Eastern European countries, participating in the International Survey of Acute Coronary Syndromes (ISACS) Archives. Over 80% of the population had traditional risk factor exposure. Age of presentation of acute coronary syndrome varied inversely with the number of traditional risk factors. The presence of four traditional risk factors was associated with up to 5-year younger age of acute coronary syndrome presentation and mortality from acute coronary syndrome. In this context, the term "premature acute coronary syndrome" was used to indicate that the patient experiences acute coronary syndrome before reaching the age that is commonly associated with acute coronary syndrome in the absence of traditional risk factors. This was, in our analysis, around 67 years for women and 63 years for men. Compared with their counterparts without the risk factor under consideration, current smoking and hypercholesterolemia were the predominant (p < 0.0001) individual-level risk factors for premature acute coronary syndrome presentation among women (RRs: 3.96; 95% CI: 3.72–4.20 and 1.31; 95% CI: 1.25–1.38, respectively) and men (RRs: 2.82; 95% CI: 2.71–2.93 and 1.39; 95% CI: 1.34–1.45, respectively). The single largest (p < 0.0001) risk factor for death from premature acute coronary syndrome was diabetes either in women (RR: 1.52; 95% CI: 1.29–1.79) or men (RR: 1.63; 95% CI: 1.41–1.89).

**Implications of all the available evidence**

Most cases of premature acute coronary syndrome and death from premature acute coronary syndrome among women and men in South Eastern Europe are attributable to a small number of modifiable risk factors. Public health policies should place significant emphasis on the four traditional risk factors and the associated lifestyle behaviors to reduce the epidemic of premature ischemic heart disease.

## Introduction

The age-standardized death rates under 65 years from ischemic heart disease are approximately twice as high in South Eastern Europe than the Western Europe average.^1^ Acute coronary syndrome is the main driver of premature mortality in ischemic heart disease. As such, acute coronary syndrome is a significant public health concern in South Eastern Europe.

The reason for the excess of premature death from acute coronary syndrome in South Eastern Europe remains hindered by insufficient information, as discrepancy in mortality may reflect region-specific differences in cardiovascular risk factors, healthcare systems, and socioeconomic factors. Yet, preventing premature acute coronary syndrome is a crucial strategy for reducing premature death associated with this condition. It follows that understanding the factors associated with premature acute coronary syndrome is essential for developing effective prevention and intervention strategies.

Potential factors associated with premature occurrence of acute coronary syndrome may include current smoking, diabetes, hypercholesterolemia, and hypertension that have been labeled as “traditional” risk factor because of the strength of the evidence supporting their role in the pathogenesis of cardiovascular disease^2^ and acute coronary syndrome.^3^ However, the relationship between these risk factors and the age of acute coronary syndrome presentation can be complex and has not been sufficiently explored. Assigning equal weight to deaths at age 80 as it does at age 60 or even at 40 years of age can mask the true burden of disease and may not accurately reflect the societal impact of these deaths including loss of productivity, financial strain and social isolation.

To address this knowledge gap, we collected data from the International Survey of Acute Coronary Syndromes (ISACS) Archives, which involves collaboration of researchers and healthcare professionals from multiple countries in South Eastern Europe. This international approach was instrumental in creating a valuable individual-level dataset from population-based cohorts while enhancing the harmonization of data collection and analysis. This standardization helps to overcome the limitation of methodological heterogeneity of currently available data sources.

## Methods

### Study design and subjects

The study population consisted of 70,953 Caucasian patients enrolled in the International Survey of Acute Coronary Syndromes (ISACS) Archives (NCT04008173) registry network for a first manifestation of acute coronary syndrome from October 2005 to January 2021 (Supplementary Figure S1). Patients with prior coronary heart disease or heart failure of unknown origin were excluded. The design of the ISACS Archives has been previously described.^4^^,^^5^ In brief, the ISACS Archives provide access to deidentified research cohorts in acute coronary syndromes.^6^ Investigators were sent a list of study-related variables with definitions and were asked to provide these data. Information about the ISACS Archives are provided in the Supplementary Methods. The hospitals that were included in the current analysis were selected on the basis of their geographic location in South Eastern Europe.

We pooled and harmonized individual-level data from 36 centers in 7 South Eastern European countries (Bosnia and Herzegovina, Croatia Kosovo, Montenegro, North Macedonia, Romania and Serbia). The local research ethics committee from each hospital approved the study. Because patient information was collected anonymously, institutional review boards waived the need for individual-informed consent. This study complies with the Declaration of Helsinki. All data were transferred to the Department of Electrical and Computer Engineering, University of California, Los Angeles, where final statistical analyses were done.

### Outcome measures

The primary outcome measure was "premature acute coronary syndrome" based on the mean age at the time of acute coronary syndrome occurrence in patients with no traditional risk factors. This approach may provide insights into the age-related patterns of acute coronary syndrome occurrence and the potential impact of traditional risk factors on the age of onset. We also measured all-cause mortality within 30 days of hospital admission in patients with premature acute coronary syndrome.

### Concomitant care and definitions

We noted the type of medications given on hospital admission and during hospitalization (fibrinolysis, antiplatelet agents [aspirin and/or P2Y_12_ inhibitors], heparins [unfractionated heparin or low-molecular-weight heparins [LMWH], glycoprotein IIb/IIIa inhibitors [GP IIb/IIIa inhibitors], statins, angiotensin-converting enzyme [ACE] inhibitors, angiotensin receptor blockers [ARBs] and β-blockers). All patients with a glomerular filtration rate <60 mL/min/1.73 m^2^ for 3 months were defined as having chronic kidney disease. Smoking habits were self-reported. Persons who were active smokers at time of the index event and smoked regularly during the previous 12 months were classified as current smokers. Former smokers were defined as those patients who had a history of smoking tobacco but were not active smokers in the last 12 months. Hypertension, hypercholesterolemia, and diabetes were assessed by designation of medical history before admission in the database. Family history of coronary artery disease was defined as death due to coronary heart disease before 55 years of age (for men) and 65 years of age (for women) in any first-degree relative or grandparent. Body mass index (BMI) was calculated as weight (kg) divided by height squared (m^2^). Patients with BMI of 30 or greater were defined as obese.

### World Health Organization and European Heart Network

Using World Bank Atlas classification 2021 of income levels, our South Eastern European cohort was constituted by patients recruited in 5 middle-income countries (MICs) with a gross national annual income (GNI) per capita between $1086 and $13,205 (Bosnia and Herzegovina, Kosovo, North Macedonia, Montenegro, and Serbia) and 2 high-income countries (HICs) with a GNI per capita of $12,536 or more (Croatia and, Romania).^7^ Ischemic heart disease age-standardised prevalence and mortality from ischemic heart disease data came from the European Cardiovascular Disease Statistics, using the 2017, or latest year available.^1^ The European Cardiovascular Disease Statistics assessed the impact of deaths under 65 on the overall mortality rate by calculating the proportion of age-standardized mortality rate under 65 years per 100,000 inhabitants in relation to the age-standardized mortality rate per 100,000 inhabitants. To examine whether this proportion varies with sex, we compared the men to women rate ratios for the proportion of death under 65 years for ischemic heart disease in relation to the overall mortality for ischemic heart disease per 100,000 inhabitants for each country. Data were expressed as percentages (Supplementary Table S1). The rate for the group of major ischemic heart disease incidence (men) was divided by the rate for the comparison group (women). A rate ratio of 1.0 indicates equal rates in the two groups, a rate ratio greater than 1.0 indicates a relative increase in risk for men, and a rate ratio less than 1.0 indicates a relative decrease in risk for men. We also illustrated the corresponding data from the World Health Organization (WHO) European Mortality Database using the 2023, or latest year available (Table 1).^8^ It can strengthen the credibility of the findings and provide more confidence in the accuracy of the reported statistics.

**Table 1:** Age-standardized mortality rates and rate ratios (women to men) for ischemic heart disease per 100,000 inhabitants, year 2023 or nearest.

Country (income group)	Men			Women			Rate ratio men to women
	Age-standardized mortality rate	Age-standardized rate under 65 years	%	Age-standardized mortality rate	Mortality rate under 65 years	%	
**South Eastern Europe**							
Bosnia and Herzegovina (MIC)	129.2	41.0	31.7	78.1	13.0	16.7	1.90
Croatia (HIC)	120.8	35.5	29.3	67.3	7.1	10.6	2.77
North Macedonia (MIC)	50.7	16.8	33.1	23.8	5.3	22.3	1.48
Montenegro (MIC)	50.9	23.3	45.8	24.8	5.4	21.8	2.10
Romania (HIC)	191.8	63.1	32.9	109.9	16.7	15.2	2.16
Serbia (MIC)	94.1	33.0	35.0	50.7	8.3	16.4	2.13
HICs	312.6	98.6	31.5	177.2	23.8	13.4	2.35
MICs	324.9	114.1	35.1	177.5	32.0	18.0	1.95
Overall	637.5	212.7	33.3	354.7	55.8	15.8	2.12
**Reference: Western Europe**							
Belgium (HIC)	46.3	12.8	27.6	19.4	3.7	19.1	1.44
France (HIC)	37.8	11.7	30.9	13.2	2.3	17.4	1.80
Germany (HIC)	88.3	20.6	23.3	39.6	4.5	11.3	2.07
Italy (HIC)	54.2	12.8	23.6	25.8	2.7	10.5	2.25
Luxembourg (HIC)	39.5	11.7	29.6	14.2	2.9	20.4	1.43
Netherlands (HIC)	34.5	8.7	25.2	15.1	2.5	16.6	1.55
Overall	300.6	78.3	26.0	127.3	18.5	14.6	1.79

Values are age-standardized rates, percentages, or rate ratios.

Abbreviations: HIC, high income country; MIC, middle income country.

### The boundaries of South Eastern Europe

The boundaries of South Eastern Europe are subject to substantial overlap and variation depending on the context in which they are used, which makes demarcation difficult.^9^^,^^10^ According to the World Factbook^11^ and the Stability Pact for South Eastern Europe,^12^ the following countries are included as “South Eastern European countries”: Albania, Bosnia and Herzegovina, Bulgaria, Croatia, Kosovo, Moldova, Montenegro, North Macedonia, Romania and Serbia. The current study refers to all European countries listed above as they were previously ruled throughout the twentieth century by socialist regimes. After the fall of socialism, these countries started a process of political, economic, and health system reform. The entire group of countries entered roughly similar directions of reform, despite differences in economic transition dynamics and the degree of government determination to implement reforms. As such, we are dealing with a relatively large group of countries that had similar starting points for cardiovascular health care transition.

### Reference for quality of care and health infrastructure

When it comes to healthcare systems in Western Europe, several countries are often considered reference points for their quality of care and health infrastructure. Historically, the Treaty of Rome set up the European Economic Community, bringing together Belgium, Germany, France, Italy, Luxembourg and the Netherlands to work together towards integration and economic growth. As such we selected those countries as reference for the Western countries in the European Cardiovascular Disease Statistics^1^ and the WHO European Mortality Database.^8^

### The definition of "premature" occurrence of acute coronary syndrome

The definition of "premature" occurrence of acute coronary syndrome can vary in different studies, and there can be confusion surrounding this definition. In general, "premature" acute coronary syndromes refers to cases of acute coronary syndrome that occur at a younger age than what is typically expected in the general population. The age threshold for defining premature acute coronary syndrome can vary, but it is often set at 55 years or younger for men and 65 years or younger for women. These age thresholds are used because atherosclerosis and related cardiovascular events tend to develop later in women than in men. For matter of simplification, the European Cardiovascular Disease Statistics set the cut-off age point at 65 years for both women and men. The same approach was used in evaluating trends in premature ischemic heart disease mortality in the United States.^13^

### Statistical analysis

Patients were categorized according to their type and number of cardiovascular risk factors. Subgroups were stratified by sex. The descriptive results were displayed by the number of traditional risk factors and age of first acute coronary syndrome. Pearson's correlation coefficient (*r*) was performed to ascertain statistical significance of trends observed. Premature hospital presentation to for first acute coronary syndrome was defined using the mean age at time of acute coronary syndrome of patients with no traditional risk factors (≤67 years in women and ≤63 years in men). The relationship between age at hospital presentation and risk factor combinations was calculated by unadjusted analyses. Given a potential concern of bias, crude data were reassessed, stratifying by each of the four risk factors. We, therefore, performed inverse probability weighting analyses to assess the association of each risk factor with premature first acute coronary syndrome and 30-day mortality from premature first acute coronary syndrome. For these analyses, we divided the risk factors into dichotomous variables and grouped patients in those with and those without the risk factor under consideration. Fixed covariates included traditional risk factors (current smoking, diabetes, hypercholesterolemia and hypertension) with the exclusion of the risk factor under consideration, other known risk factors for cardiovascular disease (family history of coronary artery disease, former smokers, obesity) and medical history of cardiovascular disease (peripheral artery disease, prior stroke).

Baseline characteristics were reported as number (percentages) for categorical variables and mean ± SD for continuous variables. We had complete data on mortality, sex, age, and index event. Patients had a missing rate on traditional risk factors ranging from 9.8% to 18.1%. We used multiple imputation by chained equations (MICE) as the imputation method to treat missing data (Supplementary Methods).^14^ To reduce the imbalance of potential confounders we used an inverse probability weighting approach on the basis of propensity scores for confounding adjustment.^15^ Because of the instability that can be induced by extreme weights, stabilized weights were used that also preserve the original sample size. We created a threshold for weights to avoid the impacts of the outliers. Specifically, we used 0.01 as threshold of the propensity weighting. Standardized differences after weighting were calculated to ensure balanced treatment groups with respect to baseline characteristics. Groups were considered balanced when the standardized difference was <10% (Supplementary Methods).^16^ We calculated the risk ratios (RRs) with their 95% CIs from logistic regression and inverse probability weighting models (Supplementary Methods). Comparisons of outcomes between groups were assessed by 2-sided p values. To further statistically compare two estimates from two different subgroup analyses, we reported the test of interaction results on the log scale.^17^ A p value <0.05 was taken to indicate that the difference between the outcomes in subgroups was unlikely to have occurred simply by chance (Supplementary Methods).

### Role of the funding source

No sponsor had any role in the design of the study or in the collection, analysis, interpretation of data, in the writing of the report, and/or in the decision to submit the paper for publication.

## Results

### Data from the WHO European Mortality Database

Table 1 presents the age-standardized mortality rates under 65 years for ischemic heart disease for most South Eastern European countries using the 2023, or latest year available. Age-standardization adjusts crude mortality rates to remove the influence of different population age structures, and hence allows more meaningful comparisons to be made between countries. The age-standardized death rates under 65 years from ischemic heart disease in South Eastern Europe are approximately twice as high than the Western Europe average. In all South Eastern European countries, age-standardized mortality rates under 65 years for ischemic heart disease are higher in men than in women. Strong geographical disparities are apparent, with relatively high rates observed in Romania (63.1 deaths per 100,000 in men and 15.2 deaths per 100,000 in women) and lower rates in North Macedonia (16.8 deaths per 100,000 in men and 5.3 deaths per 100,000 in women). Data from the WHO European Mortality Database for 2023 are confirmed by the European Cardiovascular Disease Statistics (Supplementary Table S1). There was not a clear trend of outcome rates by income levels. Whether these large differences across South Eastern European countries and among women and men reflect differences in patient characteristics, inequalities of care or, to some extent, patient selection is a matter of debate. Moreover, the European Cardiovascular Disease Statistics and the WHO European Mortality Database are limited by the fact that estimates are derived through combining data from a variety of sources including survey data, outpatient and inpatient visits of angina and first acute myocardial infarction. The reliability of some of the above reported estimates can be improved by large studies involving multiple South Eastern European countries at different economic levels and patient-level data.

### Patient-level data: the ISACS Archives cohort study

#### Baseline characteristics

The demographic features, presenting characteristics, and treatment of women and men with coronary heart disease traditional risk factors are shown in Table 2. Among patients with acute coronary syndrome, at least one of the four traditional risk factors were present in 84.2% of women and 84.6% men. For all traditional risk factors except current cigarette smoking, the prevalence was significantly higher in women than in men (Supplementary Figure S2). For fatal acute coronary syndrome (n=6097), exposure to at least one traditional risk factor ranged from 77.6% in women to 74.5% in men (Supplementary Figure S3). The prevalence and type of the four traditional risk factors changed with age (Table 3). The majority of patients with age less than 65 years (85.6% in women and 86.4% in men) had at least one traditional risk factor, with cigarette smoking being the most common in young men and hypertension in young women. Older patients had much lower rates of current cigarette smoking but typically higher rates of diabetes and hypertension, especially in women. The overall pattern of traditional risk factors existed irrespective of the geographic origin (Table 4).

**Table 2:** Baseline characteristics.

Characteristics	Women (n = 25,490)	Men (n = 45,463)	Standardized difference	p value
Age, mean (SD), years	66.4 (11.4)	60.9 (11.8)	0.47	<0.001
**Coronary heart disease risk factors**				
Diabetes	6953 (27.3)	9035 (19.9)	0.18	<0.001
Hypertension	17,665 (69.3)	27,013 (59.4)	0.21	<0.001
Hypercholesterolemia	11,083 (43.5)	18,575 (40.9)	0.05	<0.001
Current smokers	6626 (26)	21,234 (46.7)	−0.44	<0.001
Former smokers	203 (0.8)	1049 (2.3)	−0.12	<0.001
Family history of CAD	7654 (30)	13,344 (29.4)	0.01	0.06
BMI ≥30 kg/m^2^	4778 (18.7)	9260 (20.4)	−0.04	<0.001
**Clinical history of cardiovascular disorders**				
Prior stroke	1070 (4.2)	1672 (3.7)	0.03	0.001
Peripheral artery disease	582 (2.3)	1073 (2.4)	−0.01	0.52
**Clinical history of comorbidities**				
Chronic kidney disease	1381 (5.4)	1903 (4.2)	0.06	<0.001
**Clinical presentation**				
STEMI	14,689 (57.6)	28,740 (63.2)	−0.11	<0.001
NSTEMI	4987 (19.6)	8546 (18.8)	0.02	0.01
Unstable angina	5814 (22.8)	8177 (18.0)	0.12	<0.001
NSTE-ACS	10,801 (42.4)	16,723 (36.8)	0.11	<0.001
ST-segment shifts in anterior leads (at ECG)	5148 (20.2)	9915 (21.8)	−0.04	<0.001
Time to admission <12 h	18,150 (71.2)	34,486 (75.9)	−0.11	<0.001
Time to admission <2 h	4749 (18.6)	10,997 (24.2)	−0.14	<0.001
SBP at admission, mean (SD), mmHg	137.6 (29.1)	138.2 (28.3)	0.07	0.01
HR at admission, mean (SD), bpm	82.5 (20.3)	81.2 (19.6)	−0.02	<0.001
**Revascularization therapy for STEMI**				
Reperfusion therapy (fibrinolysis, PCI or CABG)	8802 (59.9)	20,577 (71.6)	−0.22	<0.001
PCI	5595 (38.1)	13,172 (45.8)	−0.16	<0.001
Fibrinolysis	3629 (24.7)	9032 (31.4)	−0.15	<0.001
Fibrinolysis and PCI	487 (3.3)	1704 (5.9)	−0.11	<0.001
CABG	229 (1.6)	445 (1.6)	−0.01	0.93
**Invasive cardiac procedures**				
PCI (all ACS)	7728 (30.3)	17,701 (38.9)	−0.18	<0.001
CABG (all ACS)	518 (2.0)	980 (2.2)	−0.01	0.27
**Medications on admission**				
Aspirin and/or P2Y_12_ inhibitors	22,885 (89.8)	41,781 (91.9)	−0.07	<0.001
Unfractionated heparin	13,960 (54.8)	25,562 (56.2)	−0.03	<0.001
LMWH	10,960 (43)	20,025 (44.1)	−0.02	0.007
Heparins (all)	21,448 (84.1)	38,919 (85.6)	−0.04	<0.001
GP IIb/IIIa inhibitors	1513 (5.9)	3525 (7.8)	−0.07	<0.001
**Medications during hospitalization and at discharge**				
β-blockers	40,714 (68.0)	5268 (47.6)	−0.07	<0.001
ACE-inhibitors/ARBs	17,075 (67)	31,592 (69.5)	−0.05	<0.001
Statins	18,449 (72.4)	35,391 (77.9)	−0.13	<0.001

Values are numbers (%) or mean (SD), unless otherwise stated.

Abbreviations: ACE, angiotensin converting enzyme; ARBs, angiotensin receptor blockers; BMI, body mass index; bpm, beats per minute; CABG, coronary artery bypass graft; CAD, coronary artery disease; CHD, coronary heart disease; ECG, electrocardiogram; GP, glycoprotein, HR, heart rate; LMWH, low molecular weight heparins; NSTE-ACS, non-ST-segment elevation acute coronary syndrome; NSTEMI, ST-segment elevation myocardial infarction; PCI, percutaneous coronary intervention; SBP, systolic blood pressure; STEMI, ST-segment elevation myocardial infarction.

**Table 3:** Prevalence of traditional coronary heart disease risk factors stratified by age and sex.

	Age <65 years		Age ≥65 years	
	Women (n = 10,203)	Men (n = 27,582)	Women (n = 15,287)	Men (n = 17,881)
**Individual CHD risk factors**				
Current smoking	4395 (43.1)	15,898 (57.6)	2231 (14.6)	5336 (29.8)
Hypercholesterolemia	4923 (48.3)	12,329 (44.7)	6160 (40.3)	6246 (34.9)
Hypertension	6639 (65.1)	15,506 (56.2)	11,026 (72.1)	11,507 (64.4)
Diabetes	2241 (22.0)	4854 (17.6)	4712 (30.8)	4181 (23.4)
**Number of CHD risk factors**				
0	1474 (14.4)	3693 (13.4)	2565 (16.8)	3328 (18.6)
1	2577 (25.3)	7750 (28.1)	4625 (30.3)	5690 (31.8)
2	3346 (32.8)	8831 (32.0)	5131 (33.6)	5513 (30.8)
3	2295 (22.5)	6057 (22.0)	2622 (17.2)	2846 (15.9)
4	511 (5.0)	1251 (4.5)	344 (2.3)	504 (2.8)

Values are numbers (%).

Abbreviation: CHD, coronary heart disease.

**Table 4:** Prevalence of traditional risk factors stratified by country.

Country (income group)	Prevalence of current smoking			Prevalence of hypercholesterolemia			Prevalence of hypertension			Prevalence of diabetes		
	Men	Women	Rate ratio men to women	Men	Women	Rate ratio men to women	Men	Women	Rate ratio men to women	Men	Women	Rate ratio men to women
Bosnia and Herzegovina (MIC)	56.5	34.6	1.63	53.0	56.7	0.93	62.8	75.0	0.84	20.6	35.6	0.58
Croatia (HIC)	46.3	27.5	1.68	47.6	50.1	0.95	67.0	81.6	0.82	19.4	27.9	0.70
North Macedonia (MIC)	48.7	28.5	1.71	25.5	24.5	1.04	60.7	70.3	0.86	17.6	26.4	0.67
Montenegro (MIC)	56.5	44.8	1.26	33.3	34.3	0.97	52.7	72.4	0.73	17.9	22.4	0.80
Kosovo (MIC)	55.7	6.9	8.07	35.7	41.4	0.86	27.1	65.5	0.41	20.0	34.5	0.58
Romania (HIC)	42.2	19.4	2.18	31.9	33.1	0.96	60.2	69.7	0.86	24.2	29.7	0.81
Serbia (MIC)	47.1	26.1	1.80	42.3	44.9	0.94	58.7	68.8	0.85	20.0	27.4	0.73
HICs	44.2	23.5	1.88	39.7	41.7	0.95	63.6	75.7	0.84	21.8	28.8	0.76
MICs	47.4	26.3	1.80	41.1	43.9	0.94	58.7	68.9	0.85	19.8	27.3	0.73
Overall	46.7	26.0	1.80	40.9	43.5	0.94	59.4	69.3	0.86	19.9	27.3	0.73

Values are percentages or rate ratio men to women.

Abbreviations: HIC, high income country; MIC, middle income country.

### Prevalence of traditional risk factors and age at hospital presentation or death from acute coronary syndrome

**Fig. 1:**
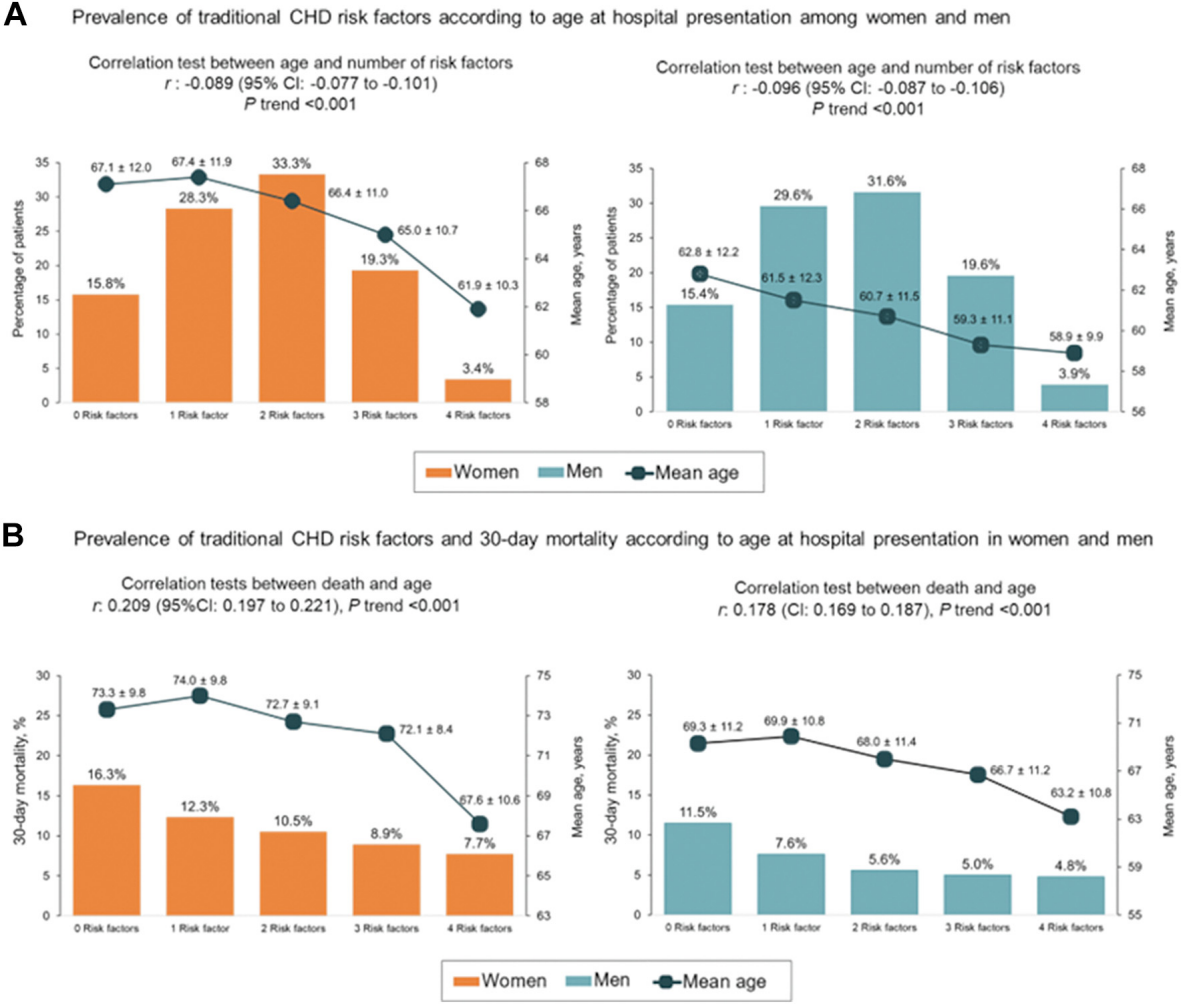
Prevalence of traditional coronary heart disease risk factors according to age at hospital presentation among women and men (panel A) and prevalence of traditional coronary heart disease risk factors and 30-day mortality according to age at hospital presentation in women and men (panel B). Abbreviation: CHD, coronary heart disease.

As the number of traditional risk factors increased, there was an inverse relationship whereby median age at time of acute coronary syndrome event declined. The presence of all four coronary heart disease risk factors significantly decreased the age of acute coronary syndrome by nearly half a decade compared with the absence of any coronary heart disease risk factors in both sexes (women: from 67.1 ± 12.0 to 61.9 ± 10.3 years; *r* = −0.089, 95% CI: −0.077 to −0.101; p < 0.001 and men: from 62.8 ± 12.2 to 58.9 ± 9.9 years; *r* = −0.096, 95% CI: −0.087 to −0.106; p < 0.001) (Fig. 1A). As well, on average, patients with four risk factors lost approximately 6 years of life compared with those with no risk factors. The age of death shifted from 73.3 ± 9.8 to 67.6 ± 10.6 years (*r* = 0.209, 95% CI: 0.197–0.221; p trend < 0.001) in women and from 69.3 ± 11.2 to 63.2 ± 10.8 years (*r* = 0.178, 95% CI: 0.169–0.187; p trend < 0.001) in men (Fig. 1B).

### Traditional risk factors and potential life expectancy free of acute coronary syndrome

**Fig. 2:**
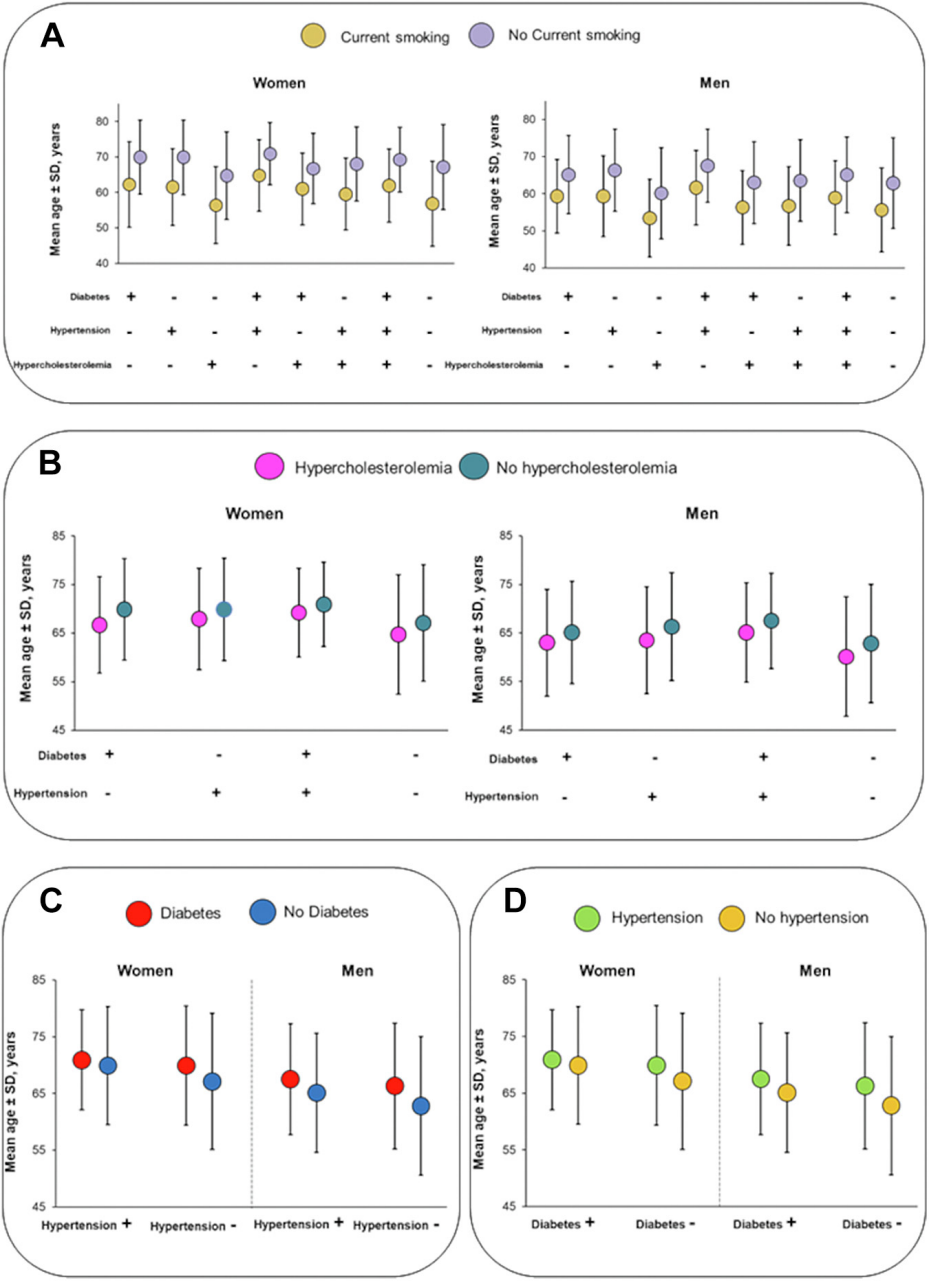
Relationship between age and traditional coronary heart disease risk factor combinations stratified by current smoking status (panel A), hypercholesterolemia (panel B), diabetes (panel C) and hypertension (panel D) among women and men. Error bars indicate standard deviations.

To estimate years of potential lifetime free of acute coronary syndrome events lost in the presence of each traditional risk factor, we compared the observed patient age at time of acute coronary syndrome event for those with and those without the risk factor under consideration (Fig. 2). On average, current smoking reduced the age at time of acute coronary syndrome event by about 7–10 years among men and women. This holds true for every risk factor combination with smoking. Similarly, in the absence of current smoking, patients with hypercholesterolemia still lost an average of 1–3 years compared with their expected life being free of hypercholesterolemia in both sexes for every risk factor combination with hypercholesterolemia. In contrast hypertension and diabetes in the absence of current smoking or hypercholesterolemia did nor negatively impact the lifetime risk for the occurrence of acute coronary syndrome.

### Traditional risk factors and premature acute coronary syndrome

**Fig. 3:**
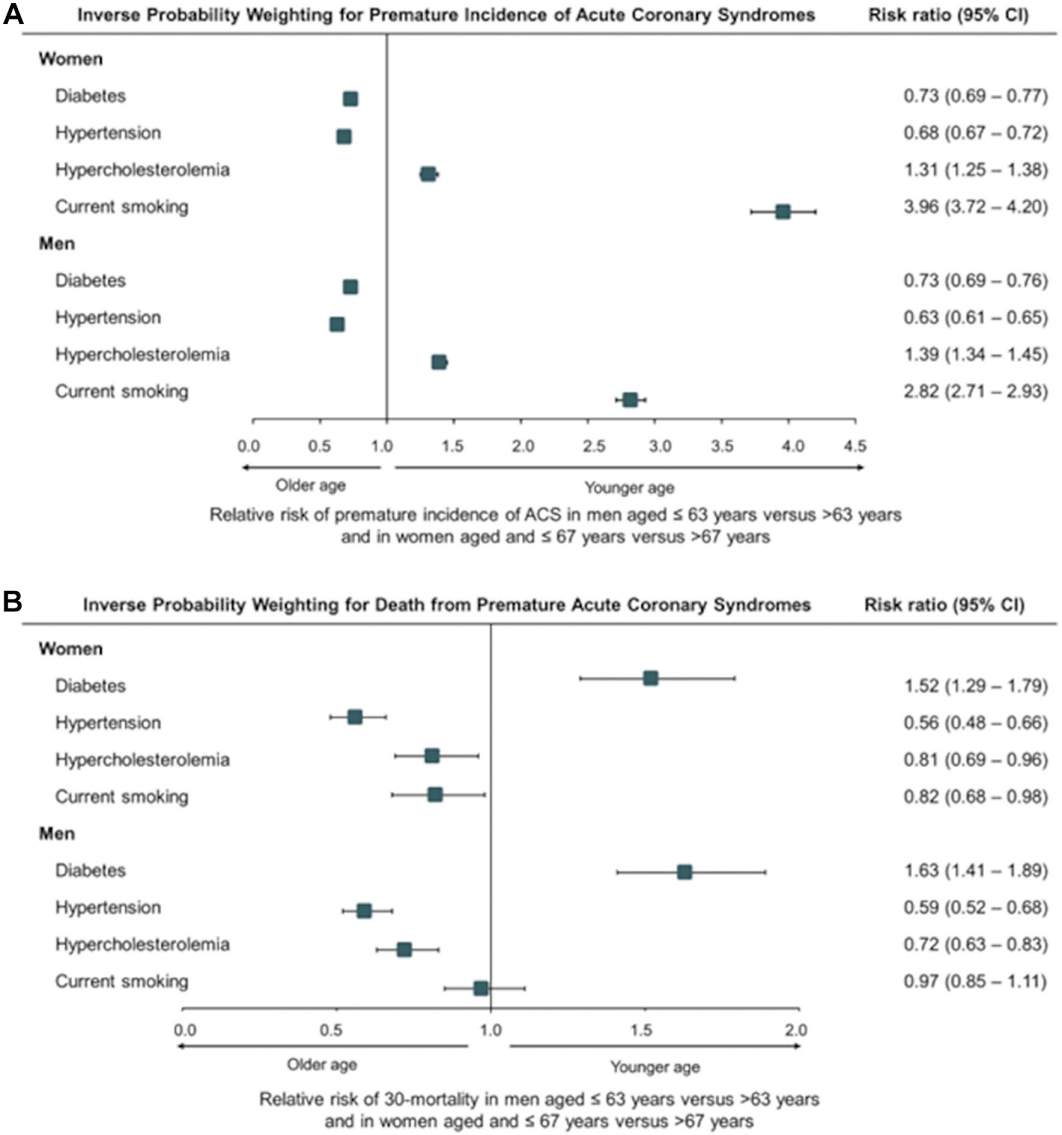
Traditional coronary heart disease risk factors and their association with premature acute coronary syndrome and death from premature acute coronary syndrome among women and men. Panel A shows risk ratios for premature incidence of acute coronary syndrome and panel B shows risk ratios for death from premature acute coronary syndrome. Risk ratios and confidence intervals were estimated with the use of inverse probability weighting with adjustment as in Table 2. Abbreviation: ACS, acute coronary syndrome.

To confirm the divergent associations that we observed in the unadjusted analyses, we used inverse probability weighting modeling (Fig. 3, Supplementary Tables S2–S17). The outcome of interest was “premature acute coronary syndrome”, which was calculated using the mean age at time of acute coronary syndrome of patients with no risk factors: ≤67 years in women and ≤63 years in men. Patients who developed an acute coronary syndrome prior to these age thresholds were considered as having suffered from “premature acute coronary syndrome”. Inverse probability weighting models showed that patients who were current smokers had approximately a three-fold increase in the risk of presenting premature acute coronary syndrome (RRs, 3.96; 95% CI: 3.72–4.20 in women and 2.82; 95% CI: 2.71–2.93 in men; p_interaction_ < 0.001). The corresponding RRs for patients with hypercholesterolemia were 1.31 (95% CI: 1.25–1.38) in women and 1.39 (95% CI: 1.34–1.45) in men (p_interaction_ = 0.03). Diabetes was most strongly associated with death from premature acute coronary syndrome (RRs: 1.52; 95% CI: 1.29–1.79 in women; 1.63; 95% CI: 1.41–1.89 in men; p_interaction_ = 0.27). Hypertension was not predictive of premature acute coronary syndrome or death from premature acute coronary syndrome. Comparisons of RRs by interaction test are reported in Supplementary Tables S18–S20.

## Discussion

The current study demonstrates that there are major differences in traditional risk-factor burden that translate into marked differences in acute coronary syndrome-free survival and subsequent premature mortality for acute coronary syndrome among men and women in South Eastern Europe. This assumes particular importance in the presence of all four traditional risk factors, which is consistently associated with up to 5-year younger age of acute coronary syndrome presentation and mortality from acute coronary syndrome. Current smoking and hypercholesterolemia had a major impact on acute coronary syndrome-free survival and diabetes was a significant risk factor for premature death. This overall pattern was largely independent of sex and geographic region. Public health policies should place significant emphasis on the four traditional risk factors and the associated lifestyle behaviors to reduce the epidemic of premature ischemic heart disease in South Eastern Europe. These observations raise significant clinical issues.

Because atherosclerosis typically develops later in women than in men, high risk factor prevalence in women would lead to the development of acute coronary syndrome at older age as in men with comparable risk factor burden. This hypothesis is substantiated by the results of the current study. The presence of all four coronary heart disease risk factors significantly decreased the age of hospital presentation for acute coronary syndrome and subsequent mortality on average by nearly half a decade compared with the absence of any coronary heart disease risk factors in both sexes. Among men the mean age of acute coronary syndrome free survival declined from 63 to 59 years whereas among women the risk rose with older age, but still the presence of all four coronary heart disease risk factors shortened mean acute coronary syndrome free survival by 5 years, specifically from 67 to 62 years. In essence, patients with traditional risk factors have a significant survival disadvantage compared with those without risk factors.

A critically important feature of traditional risk factors is that each has a quantitively different impact on the development of premature acute coronary syndrome. Cigarette smoking played a critical role in reducing the age of acute coronary syndrome presentation with current smokers having a three-fold increase in the risk of premature acute coronary syndrome compared with their counterparts. A significant decrease in the age of acute coronary syndrome presentation was also observed when patients showed a history of hypercholesterolemia. The estimated effect of hypercholesterolemia was approximately a 30% increase in risk of premature acute coronary syndrome suggesting the contribution of hypercholesterolemia as a major risk enhancer for early atherosclerosis. By contrast, we did not find significant gains in acute coronary syndrome free survival with hypertension and diabetes. These findings are concordant with recent observations from a prospective cohort of individuals aged 45 years or less presenting with stable obstructive coronary artery disease, for whom hypertension and diabetes were rare, but active smoking and hypercholesterolemia were much more frequent.^18^

Our data should not be interpreted as indicating that diabetes or hypertension have no overall effect on cardiovascular disease or health in general, because the data reported in the current investigation focused only on the occurrence of acute coronary syndrome as first manifestation of coronary heart disease. Thus, our estimates are not designed for clinicians to prioritize one prevention treatment over the other because cardiovascular disease outcomes and time of occurrence of outcomes vary substantially based on patients’ individual burden of risk factors and the risk factors’ differential effects on various manifestations of cardiovascular disease.^19^ As such, we acknowledge that failure of hypertension and diabetes to impact acute coronary syndrome free survival may be likely attributable to a survivor bias because patients with the above risk factors may die at a much younger age than that expected to occur with acute coronary syndrome in the context of other competing risks for other manifestations of cardiovascular disease and death. Hypertension is associated with a substantially higher risk for heart failure and stroke^20^^,^^21^ and is particularly prevalent among younger stroke patients.^22^ As well, diabetes is associated with a wide range of incident cardiovascular diseases, but heart failure and peripheral arterial disease are the most common initial manifestations of cardiovascular disease.^23^ Moreover, several studies have shown that diabetes is associated with out-of-hospital cardiac arrest and sudden cardiac death.^24^ According to these observations, we found that diabetes was highly predictive of death in patients with premature acute coronary syndrome suggesting that it accounts for a large variance in survival from acute coronary syndrome. In summary, the occurrence of acute coronary syndrome and death from acute coronary syndrome are distinct matters, and it is important to differentiate between them. The risk factors for the occurrence of premature acute coronary syndrome may be different from those contributing to the risk of death from premature acute coronary syndrome. While current smoking, and high cholesterol levels are risk factors for a premature occurrence of acute coronary syndrome, the severity of the event and the presence of other complications may play a more significant role in determining the risk of death from premature acute coronary syndrome, which is the case of those patients with diabetes who experience a premature acute coronary syndrome.

Our findings have important implications for ischemic heart disease prevention and public health practice worldwide and, much more, in South Eastern Europe where the risk-factor burden and the incidence of cardiovascular disease are among the highest in Europe. Despite the fact that the risks of developing and dying from acute coronary syndrome are substantially higher than those for cancer, surveys continue to indicate that most people perceive that the risk of death from cancer is higher than that of cardiovascular disease.^25^ People perceive cancer as something that inevitably reduce the length of their life. By contrast, they believe that the new advances in treatment of cardiovascular disease are lifesaving and would not reduce the length of their life. Thus, a substantial deficit in awareness of the relation between traditional risk factors as precursor of acute coronary syndrome and shorter survival or lower quality of life exists. As such, the data of the current analysis underscore the importance of estimating and communicating lifetime risk in public health campaigns related to coronary heart disease.

Premature acute coronary syndrome can, indeed, be socially devastating, both for the affected individuals and their broader social circle. People experiencing premature acute coronary syndrome may face permanent disability, which may result in a loss of income, financial strain, and reduced economic stability for the individual and their family.^26^ Medical expenses related to the treatment of acute coronary syndrome can be substantial. For individuals without adequate health insurance or financial resources, survival from acute coronary syndrome may result in remarkably financial hardship.^27^ Physical limitations and the concern about the possibility of a recurrence of acute coronary syndrome can lead to social isolation, as individuals may become less active and engaged in social activities.^28^ In summary, while the ultimate goal is to prevent acute coronary syndrome at all ages, acknowledging the age at which acute coronary syndrome occurs is essential for tailoring strategies and interventions to specific populations. A focus on premature acute coronary syndrome aligns with the goal of improving public health policies aimed at preventing acute coronary syndrome in South Eastern European countries. Public health strategies can promote regular check-ups and risk factor management.

This study has some limitations. This study was observational in nature and is therefore subject to potential confounding. Inverse probability weighting analyses minimized such confounding. Residual confounding due to diet, physical activity, stress, and environmental factors might be a potential source of bias. We did not have a control group without acute coronary syndrome for comparison. However, previous work has shown that exposure to one or more of the traditional risk factors is also highly prevalent among individuals who do not develop clinical coronary heart disease.^29^ Exposure to the etiologic agents for coronary heart disease is, therefore necessary but not sufficient to cause acute clinical manifestation of the disease. Additionally, some of the risk factors were ascertained by the general practitioner, which might have led to errors in the dataset. However, the true prevalence of the traditional risk factors is certainly higher than that identified in our study, as approximately 30% of patients with hypertension, hypercholesterolemia and diabetes are unaware that they have these risk factors.^30^^,^^31^ Thus, detailed assessment for risk factors would almost certainly lead to higher prevalence rates than those reported in the current study. Familial hypercholesterolemia was not considered in our analysis because of the lack of available data to calculate the Dutch Lipid Clinic Network score.^32^^,^^33^ Its prevalence can vary significantly depending on the population being studied. In our study population, approximately 30% of individuals had a positive family history of coronary artery disease, which may suggest a high burden of familial hypercholesterolemia and premature coronary disease development. We acknowledge that we lack information on medications given prior to the index event. Prior medications may have decreased the discriminatory power of traditional risk factors to accurately predict acute coronary syndrome. Nonetheless, the choice of including only patients with first acute coronary syndrome reduces the possibility that our population might have substantially altered lifestyles or risk factor levels before the index event. We also acknowledge that information about modifiable risk factors was available from the baseline records, and the effect of changes in exposure over time are not known. Moreover, caution is needed in interpreting our data as being representative of each country. However, risk-factor levels in our study cohort of acute coronary syndrome parallel those observed in other regions of the world when recruiting patients with ischemic heart disease, which provides potential replication.^34^ Therefore, we think that there is little material bias in our results despite the use of a retrospective design. Finally, our study consisted solely of a population of European white patients. Application of these absolute lifetime risk estimates to other race/ethnic groups is uncertain. The categorization of geographic regions by the World Factbook and the Stability Pact for South Eastern Europe was adapted to accommodate cohort size and representativeness of a geographic region, so a different categorization of regions may produce different results.

### Conclusion

Most cases of premature acute coronary syndrome and death from premature acute coronary syndrome among women and men in South Eastern Europe are attributable to a small number of modifiable risk factors. Public health policies should place significant emphasis on the four traditional risk factors and the associated lifestyle behaviors to reduce the epidemic of premature ischemic heart disease. Awareness of shorter life expectancy free of acute coronary syndrome in the presence of traditional risk factors may motivate lifestyle changes and adherence to preventive therapy both in women and men.

## Contributors

RB and EC contributed to study design, data interpretation, literature search and writing and editing of the manuscript. JY contributed to data analysis, and data interpretation. MB contributed to the literature search and editing of the manuscript. ZV contributed to data collection and editing of the manuscript. GM contributed to editing of the manuscript. MZ, MV, SK and DM contributed to data collection and editing of the manuscript. OM contributed to data interpretation and editing of the manuscript. LB contributed to data interpretation and editing of the manuscript.

## Data sharing statement

To guarantee the confidentiality of personal and health information, only the authors have had access to the data during the study. The source codes for this manuscript are uploaded on GitHub.

## Declaration of interests

None.
