# Supplementary material for: Traditional risk factors and premature acute coronary syndromes in South Eastern Europe: a multinational cohort study
Source: Lancet Reg Health Eur. 2024 Jan 2;38:100824. doi: 10.1016/j.lanepe.2023.100824 (PMC10928297; doi:10.1016/j.lanepe.2023.100824)
Supplement: Supplementary Material [file mmc1.docx]

**Supplementary Data**

**Traditional risk factors and premature acute coronary syndromes in South Eastern Europe: a multinational cohort study.**

**Table of contents**

[**SUPPLEMENTARY METHODS** 2](#_Toc151570323)

[**The International Survey of Acute Coronary Syndromes (ISACS) Archives**. 2](#_Toc151570324)

[**Information on Registries of the ISACS- Archives** 2](#_Toc151570325)

[**Definition of risk factors for coronary heart disease** 3](#_Toc151570326)

[**Multiple Imputation using Chained Equation (MICE) algorithm.** 3](#_Toc151570327)

[**Inverse Propensity Score Weighting Analysis** 3](#_Toc151570328)

[**Computation of Relative Risk and its Confidence Interval** 4](#_Toc151570329)

[**Comparison of means and prevalences in the weighted sample** 6](#_Toc151570330)

[**Interaction test** 6](#_Toc151570331)

[**SUPPLEMENTARY RESULTS** 7](#_Toc151570332)

[**Interaction tests** 7](#_Toc151570333)

[**Figure S1.** Study flow chart 8](#_Toc151570334)

[**Figure S2.** Prevalence of traditional risk factors for coronary heart disease among men and women hospitalized for acute coronary syndromes 9](#_Toc151570335)

[**Figure S3.** Prevalence of traditional risk factors for coronary heart disease among men and women with fatal acute coronary syndromes 10](#_Toc151570336)

[**Table S1.** Age-standardized rate ratios (women to men) for ischemic heart disease per 100,000 inhabitants 11](#_Toc151570337)

[**Table S2.** Clinical factors and premature incidence of acute coronary syndrome stratified by smoking status in women: Inverse probability weighting 13](#_Toc151570338)

[**Table S3.** Clinical factors and premature incidence of acute coronary syndrome stratified by hypercholesterolemia status in women: Inverse probability weighting 14](#_Toc151570339)

[**Table S4.** Clinical factors and premature incidence of acute coronary syndrome stratified by diabetes status in women: Inverse probability weighting 15](#_Toc151570340)

[**Table S5.** Clinical factors and premature incidence of acute coronary syndrome stratified by hypertension status in women: Inverse probability weighting 16](#_Toc151570341)

[**Table S6.** Clinical factors and premature incidence of acute coronary syndrome stratified by smoking status in men: Inverse probability weighting 17](#_Toc151570342)

[**Table S7.** Clinical factors and premature incidence of acute coronary syndrome stratified by hypercholesterolemia status in men: Inverse probability weighting 18](#_Toc151570343)

[**Table S8.** Clinical factors and premature incidence of acute coronary syndrome stratified by diabetes status in men: Inverse probability weighting 19](#_Toc151570344)

[**Table S9.** Clinical factors and premature incidence of acute coronary syndrome stratified by hypertension status in men: Inverse probability weighting 20](#_Toc151570345)

[**Table S10.** Clinical factors and death from premature acute coronary syndrome stratified by smoking status in women: Inverse probability weighting 21](#_Toc151570346)

[**Table S11.** Clinical factors and death from premature acute coronary syndrome stratified by hypercholesterolemia status in women: Inverse probability weighting 22](#_Toc151570347)

[**Table S12.** Clinical factors and death from premature acute coronary syndrome stratified by diabetes status in women: Inverse probability weighting 23](#_Toc151570348)

[**Table S13.** Clinical factors and death from premature acute coronary syndrome stratified by hypertension status in women: Inverse probability weighting 24](#_Toc151570349)

[**Table S14.** Clinical factors and death from premature acute coronary syndrome stratified by smoking status in men: Inverse probability weighting 25](#_Toc151570350)

[**Table S15.** Clinical factors and death from premature acute coronary syndrome stratified by hypercholesterolemia status in men: Inverse probability weighting 26](#_Toc151570351)

[**Table S16.** Clinical factors and death from premature acute coronary syndrome stratified by diabetes status in men: Inverse probability weighting 27](#_Toc151570352)

[**Table S17.** Clinical factors and death from premature acute coronary syndrome stratified by hypertension status in men: Inverse probability weighting 28](#_Toc151570353)

[**Table S18.** Interaction test for comparing two estimated risk ratios (women vs men) : incidence of premature acute coronary syndrome with current smoking: 29](#_Toc151570354)

[**Table S19.** Interaction test for comparing two estimated risk ratios (women vs men): incidence of premature acute coronary syndrome with hypercholesterolemia: 30](#_Toc151570355)

[**Table S20.** Interaction test for comparing two estimated risk ratios (women vs men): incidence of death from premature acute coronary syndrome with diabetes: 31](#_Toc151570356)

[**REFERENCES** 32](#_Toc151570357)

# **SUPPLEMENTARY METHODS**

## **The International Survey of Acute Coronary Syndromes (ISACS)** **Archives**.

The ISACS Archives network (NCT04008173) is part of ISACS (NCT01218776) health care program. It is a collaborative network of research centers that support rapid development of new scientific information and analytic tools. The ISACS Archives uses an established informatics infrastructure, hosted and managed by the ISACS TC registry (NCT01218776) and the Department of Electrical and Computer Engineering, University of California, Los Angeles, which enables sharing of data. The ISACS Archives includes sites in which investigators are committed to collecting good-quality data without a strict proportionate sampling. Registries enrolled in the ISACS Archives use data definition for the measures/experiments that are harmonized to the standard variables of the ISACS-TC^1^ Participation in the research network does not eliminate the ability of any individual patient registry from analyzing only the data from the registry alone.

## **Information on Registries of the ISACS-** **Archives**

Data Sharing Procedures Data submitted to the ISACS ARCHIVES must be de-identified by the investigator prior to submission. Any problematic data items will be summarized in a report and returned to the investigator for corrective action, if necessary, before data are accepted. Investigators will be able to gain access to ISACS ARCHIVES data by submitting a data access request to ISACS-TC (NCT01218776) contact persons.

The ISACS-Archives study, collected data from 41 centers in 12 European countries: Bosnia and Herzegovina, Croatia, Italy, Kosovo, Lithuania, Macedonia, Hungary, Moldova, Montenegro, Romania, Russian Federation, and Serbia. Among these sites, there were 22 tertiary health care services providing percutaneous coronary intervention (PCI). In the current analysis, we pooled and harmonized individual-level data from 36 centers in 7 South Eastern European countries (Bosnia and Herzegovina, Croatia Kosovo, Montenegro, North Macedonia, Romania and Serbia). Vital status was available in 100% of participants.

## **Definition of risk factors for coronary heart disease**

Smoking habits were self-reported. Persons who were active smokers at time of the index event and smoked during the previous 12 months were classified as current smokers. Former smokers were defined as those patients who had a history of smoking tobacco but were not active smokers in the last 12 months. Hypertension, hypercholesterolemia, and diabetes were assessed by designation of medical history before admission in the database. Family history of coronary artery disease was defined as death due to coronary heart disease before 55 years of age (for men) and 65 years of age (for women) in any first-degree relative or grandparent. Body mass index (BMI) was calculated as weight (kg) divided by height squared (m^2^). Patients with BMI of 30 or greater were defined as obese.

**Multiple Imputation using Chained Equation (MICE) algorithm.**

Multiple Imputation using Chained Equation (MICE) algorithm is an efficient and popular method to fill in missing data where each missing value on some records is replaced by a value obtained from related cases in the whole set of records. Thus, imputation for clinical features was conducted using the chained equations across other features.^3^ More specifically, MICE algorithm sequentially imputes the missing values of clinical features based on both observed values and previously imputed values. This sequential imputation is conducted via chained equations.

We tried multiple imputations using the MICE algorithm for the initial analyses to address the uncertainty in the imputation process. More specifically, we generated multiple imputed datasets and checked whether the conclusions were consistent across the different imputed datasets. If the conclusions were consistent across multiple imputed datasets, we used a single imputed dataset (by MICE algorithm) as the final dataset to report the results of statistical analyses in the paper.

**Inverse Propensity Score Weighting Analysis**

We used Inverse Propensity Score Weighting (IPW) to balance the distribution of covariates between two patient groups. Note that we use Logistic Regression to estimate the propensity scores ({P}(Z=1 | x)). If *e* denotes the estimated propensity score (i.e. e=\hat{P}(Z=1 | x), where the patient x is included in patient group 1; then, 1-e = \hat{P}(Z=0 | x)), then the original sample is weighted by the following weights: Z/e+(1−Z)/ 1−e where Z represents the patient group. For instance, women (Z=1) are assigned a weight equal to the reciprocal of the propensity score (1/e), while men (Z=0) are assigned a weight equal to the reciprocal of one minus the propensity score (1/1-e). The weighting procedure for each sample balances the covariate distributions between two patient groups.^4^

Inverse probability weighting method can potentially result in unstable and biased estimates if some of the weights are very high. To avoid excessive weights, we compared results with other methods for handling confounding. We included variables in a multivariable. We also used XGBoost, a decision-tree-based ensemble machine learning algorithm, as an alternative multivariable model for estimating the probability of treatment. Conclusions from theses analyses were the same as our current results. Further, we created a threshold for weights to avoid the impacts of the outliers (we use 0.01 as threshold). Therefore, the inverse probability weighting analyses presented in the current analysis were quite stable.

## **Computation of Relative Risk and its Confidence Interval**

In a two-group cohort study, the risk ratio (RR, also called relative risk), is usually applied to compare risks of a health event between two independent binomial populations that differ by a demographic characteristic (i.e. sex, age) or by the level of exposure to a specific drug or risk factor. In such types of studies, data can be summarized in a confusion matrix as follows:

|  | **Risk of Designated Outcome** | |  |
| --- | --- | --- | --- |
|  | **Yes** | **No** | **Total** |
| **Exposed** | a | b | a+b (*H_1_*) |
| **Unexposed** | c | d | c+d (*H_0_*) |
| **Total** | a+c | b+d |  |

Where *H_1_* and *H_0_* correspond to the total number of exposed and unexposed patients, respectively, whereas *a and c* represent the number of exposed and unexposed patients at risk for the designated outcome, respectively.

RR is defined as the ratio between the risk of outcome in exposed patients (*H_1_*) and the risk of outcome in unexposed patients (*H_0,_*) which can be summarized as:

$$RR=\frac{\left( \frac{a}{H_{1}} \right)}{\left( \frac{c}{H_{0}} \right)}$$

When applying this equation to an IPW balanced population, $\frac{a}{H_{1}}$ will be assigned a weight equal to the reciprocal of the propensity score ($\frac{1}{e}$) and $\frac{c}{H_{0}}$ will be weighted by the reciprocal of one minus the propensity score ($\frac{1}{(1-e)}$).

In order to compute the lower and upper (1-α) confidence limit RR_L_ for RR, we operate in the assumption of log normal distribution^5^. In particular, the variate $\log\frac{\left( \frac{a}{H_{1}} \right)}{\left( \frac{c}{H_{0}} \right)}$= $\log\frac{a}{H_{1}}- \log\frac{c}{H_{0}}$is approximately normally distributed with approximate mean log(RR) and estimated variance $\frac{1-\left( \frac{a}{H_{1}} \right)}{a}$ + $\frac{1-(\frac{c}{H_{0}})}{c}$ .

It follows that RR_L_ can be computed by solving the following equation:

$$\frac{\left[ log( \frac{\frac{a}{H_{1}}}{\frac{c}{H_{0}}})- \log({RR}_{L}) \right]}{\left[ \frac{1-\left( \frac{a}{H_{1}} \right)}{a} + \frac{1-(\frac{c}{H_{0}})}{c} \right]^{1/2}}=z_{1-\alpha}$$

Where $z_{1-\alpha}$, is the 100(1-α) percentage point of the N(O, 1) distribution

## **Comparison of means and prevalences in the weighted sample**

To evaluate the balance of the baseline covariate distributions between treatment and control groups, standardized difference (SD) is widely used in inverse probability weighting (IPW) framework. For the baseline analysis, we use standard SD which is defined as follows: $\frac{m_{t}-m_{c}}{\sqrt{\frac{s_{t}^{2}+s_{c}^{2}}{2}}}$ for continuous variables and $\frac{m_{t}-m_{c}}{\sqrt{\frac{m_{t}(1-m_{t})+m_{c}(1-m_{c})}{2}}}$ for binary variables where $m_{t}, m_{c}$ are sample mean of the variables for treatment and control group, and $s_{t}^{2}, s_{c}^{2}$ are sample variance of the variables for treatment and control group, respectively. For IPW analysis, we use weighted SD where $m_{t}, m_{c}$ are replaced with weighted sample mean of the variables for treatment and control group, and $s_{t}^{2}, s_{c}^{2}$ are replaced with weighted sample variance of the variables for treatment and control group, respectively. Weights are determined by the inverse probability of treatment received. In general, 0.1 is the reasonable threshold to determine whether two distributions are balanced (i.e., if SD >0.1, the baseline covariate is imbalanced).^6^

**Interaction test**

The comparison of two estimated quantities, each with its standard error, is a general method that can be applied widely. We compared the risk ratios of 30-day mortality from two subgroups (women versus men) stratified by the presence of traditional risk factors (traditional coronary heart disease risk factors versus no traditional coronary heart disease risk factors. These measures were always analyzed on the log scale because the distributions of the log ratios tend to be closer to normal than of the ratios themselves. If the estimates are *E*1 and *E*2 with standard errors SE(*E*1) and SE(*E*2), then the difference *d*=*E*1 - *E*2 has standard error SE(*d*)=Ö[SE(*E*1)2 + SE(*E*2)2] i.e., the square root of the sum of the squares of the separate standard errors. The ratio *z*=*d*/SE(*d*) gives a test of the null hypothesis that in the population the difference *d* is zero, by comparing the value of *z* to the standard normal distribution. The 95% confidence interval (CI) for the difference is *d*-1.96SE(*d*) to *d*+1.96SE(*d*).^7^

# **SUPPLEMENTARY RESULTS**

## **Interaction tests**

We tested **(Table S17)** whether there was a significant interaction between sex (women versus men) and current smoking (current smoking versus no current smoking) in function of the outcome (premature ACS)**.** We obtained the logs of the risk ratios and their confidence intervals (rows 2 and 4). As 95% confidence intervals were obtained as 1.96 standard errors (SE) on either side of the estimate, the SE of each log relative risk was obtained by dividing the width of its confidence interval by 2×1.96 (row 6). The estimated difference in log relative risks was *d*=E1- E2= 0.34 (row 7) and its standard error 0.07 (row 8). From these two values, we tested the interaction and estimated the ratio of the relative risks (with confidence interval). The test of interaction was the ratio of *d* to its standard error: z=9.22, which gave a *P* value<0.001 when we referred it to a table of the normal distribution (row 10). The estimated interaction effect was exp =1.40 (row 11). The confidence interval for this effect was 0.27 to 0.41 on the log scale (row 9). Transforming back to the relative risk scale, we got 1.31 to 1.51 (row 12). The same approach was used for comparing the women to men risk ratios for premature acute coronary syndrome in presence of hypercholesterolemia and for comparing the women to men risk ratios for death from premature acute coronary syndrome in presence of diabetes **(Table S18 and S19).**

## **Figure S1.** Study flow chart


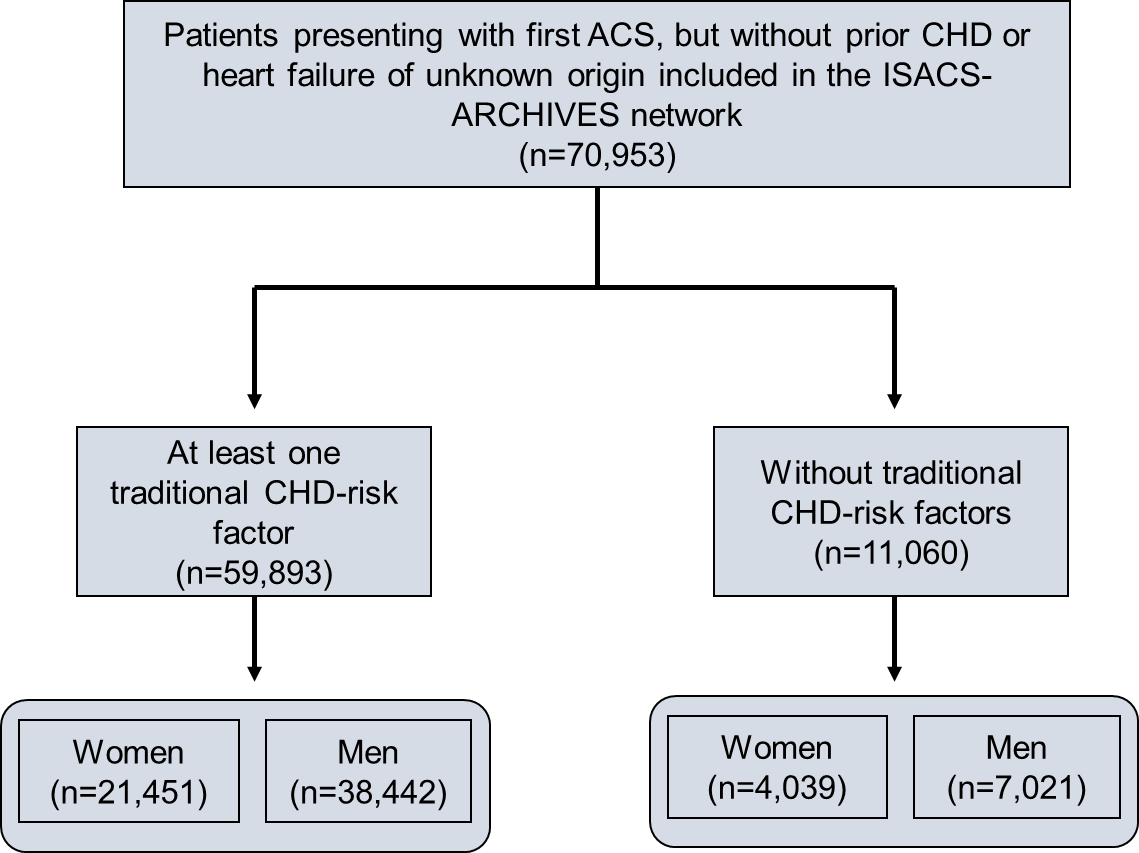


**Abbreviations:** ACS, acute coronary syndromes; CHD, coronary heart disease; NSTE-ACS, non-ST-segment elevation acute coronary syndromes; STEMI, ST-segment elevation acute coronary syndromes

## **Figure S2.** Prevalence of traditional risk factors for coronary heart disease among men and women hospitalized for acute coronary syndromes


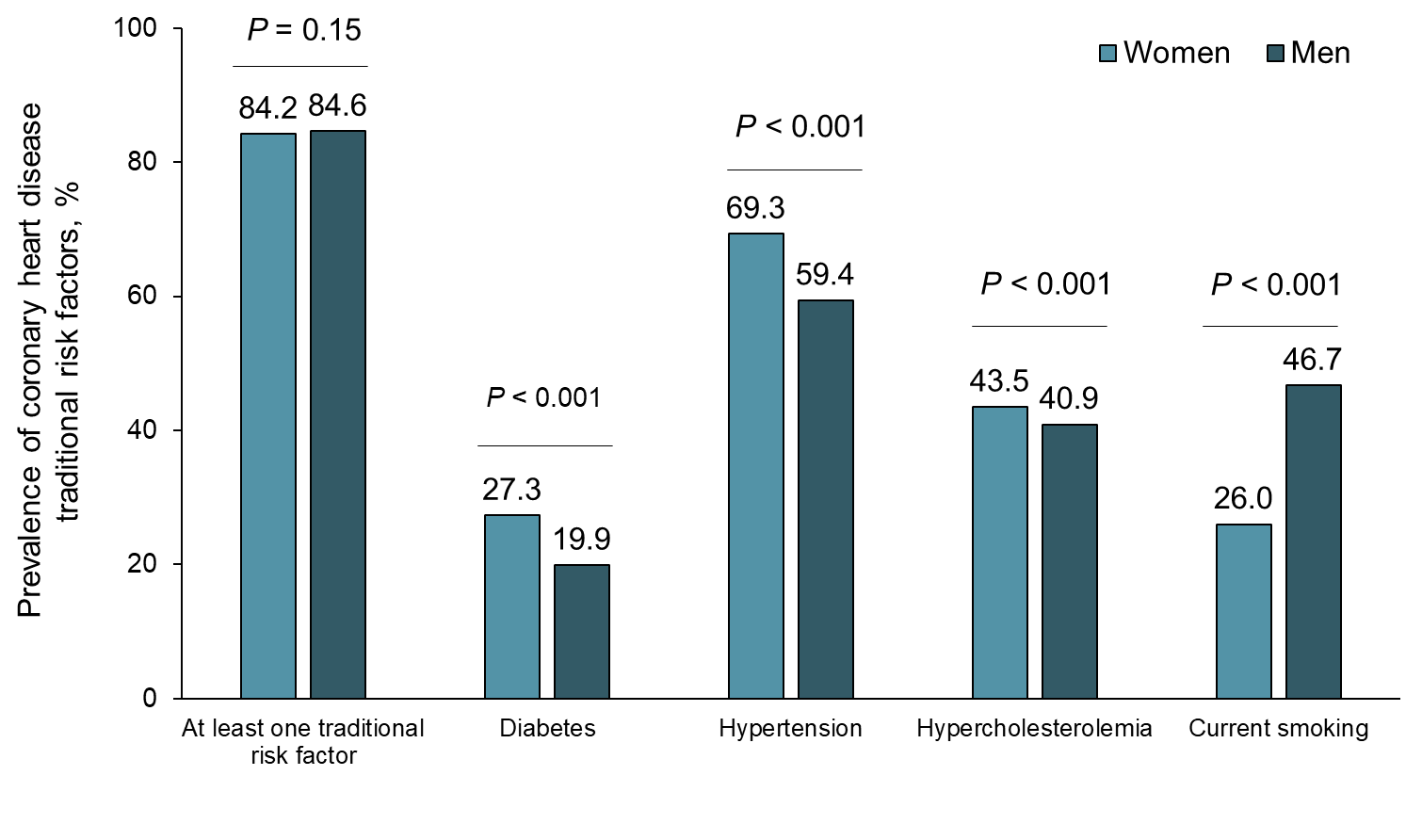


**Figure S3.** Prevalence of traditional risk factors for coronary heart disease among men and women with fatal acute coronary syndromes


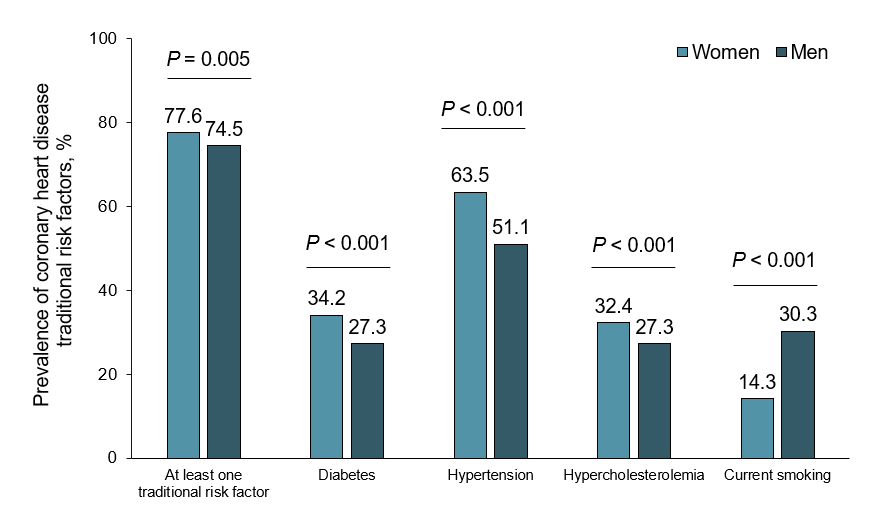


| **Table S1.** Age-standardized rate ratios (women to men) for ischemic heart disease per 100,000 inhabitants | | | | | | | | |
| --- | --- | --- | --- | --- | --- | --- | --- | --- |
| **Country (income group)** | **Men** | | |  | **Women** | | | **Rate ratio**  **men to women** |
|  | **Age-standardized mortality rate** | **Age-standardized mortality rate under 65 years** | **%** |  | **Age-standardized mortality rate** | **Age-standardized mortality rate under 65 years** | **%** |  |
| **South Eastern Europe** |  |  |  |  |  |  |  |  |
| Bosnia and Herzegovina (MIC) | 185 | 37 | 20.0 |  | 132 | 10 | 7.5 | 2.66 |
| Croatia (HIC) | 341 | 47 | 13.8 |  | 244 | 10 | 4.0 | 3.45 |
| North Macedonia (MIC) | 188 | 44 | 23.4 |  | 103 | 15 | 14.6 | 1.60 |
| Montenegro (MIC) | 138 | 30 | 21.7 |  | 72 | 12 | 16.7 | 1.30 |
| Romania (HIC) | 364 | 57 | 15.6 |  | 250 | 16 | 6.4 | 2.43 |
| Serbia (MIC) | 194 | 39 | 20.1 |  | 125 | 11 | 8.9 | 2.26 |
| HICs | 705 | 104 | 14.7 |  | 494 | 26 | 5.2 | 2.82 |
| MICs | 705 | 150 | 21.2 |  | 432 | 48 | 11.1 | 1.91 |
| Overall | 1,410 | 254 | 18.0 |  | 926 | 74 | 9.7 | 1.85 |
| **Reference: Western Europe** |  |  |  |  |  |  |  |  |
| Belgium (HIC) | 113 | 16 | 14.2 |  | 51 | 4 | 7.8 | 1.81 |
| France (HIC) | 77 | 12 | 15.6 |  | 32 | 2 | 6.3 | 2.49 |
| Germany (HIC) | 189 | 22 | 11.6 |  | 101 | 5 | 5.0 | 2.35 |
| Italy (HIC) | 148 | 16 | 10.8 |  | 83 | 4 | 4.8 | 2.24 |
| Luxembourg (HIC) | 115 | 20 | 17.4 |  | 45 | 2 | 4.4 | 3.91 |
| Netherlands (HIC) | 90 | 11 | 12.2 |  | 45 | 3 | 6.7 | 1.83 |
| Overall | 732 | 97 | 13.3 |  | 357 | 20 | 5.6 | 2.37 |
| **Abbreviations:** IHD=ischemic heart disease; HIC=high income country; MIC=middle income country.  Source of data: European Cardiovascular Disease Statistics 2017, European Heart Network^8^ | | | | | | | | |

| **Table S2.** Clinical factors and premature incidence of acute coronary syndrome stratified by smoking status in women: Inverse probability weighting | | | |
| --- | --- | --- | --- |
|  | **Women** | |  |
| **Characteristics** | **Current Smokers**  **(n = 6,626)** | **Non**  **Current Smokers**  **(n = 18,864)** | **Standardized difference** |
| **Coronary heart disease risk factors** | | | |
| Diabetes | 27.8 | 27.1 | 0.0169 |
| Hypertension | 69.4 | 69.2 | 0.0038 |
| Hypercholesterolemia | 43.2 | 43.5 | -0.0061 |
| Family history of CAD | 30.1 | 30.3 | -0.0040 |
| Former smokers | 0.0 | 0.8 | -0.0861 |
| BMI >30 kg/m^2^ | 18.9 | 18.8 | 0.0023 |
| **Clinical history of CVD** |  |  |  |
| PAD | 2.2 | 2.3 | -0.0087 |
| Prior stroke | 4.1 | 4.2 | -0.0044 |
| **Outcomes** |  |  | ***P* value** |
| Premature ACS (≤67years) presentation | 70.1 | 37.2 | <0.001 |
| Risk Ratio (95% CI) | 3.96 (3.72 – 4.20) | |  |
| Data are presented as percentages (%) or mean ± standard deviation, unless otherwise specified.  **Abbreviations:** ACS, acute coronary syndrome; BMI, body mass index; CAD, coronary artery disease; CVD, cardiovascular disease; PAD, peripheral artery disease. | | | |

| **Table S3.** Clinical factors and premature incidence of acute coronary syndrome stratified by hypercholesterolemia status in women: Inverse probability weighting | | | |
| --- | --- | --- | --- |
|  | **Women** | |  |
| **Characteristics** | **Hypercholesterolemia (n = 11,083)** | **No-**  **Hypercholesterolemia**  **(n = 14,407)** | **Standardized difference** |
| **Coronary heart disease risk factors** | | | |
| Diabetes | 27.8 | 27.3 | 0.0107 |
| Hypertension | 70.4 | 69.5 | 0.0181 |
| Current Smokers | 27.2 | 26.6 | 0.0136 |
| Family history of CAD | 30.4 | 30.3 | 0.0038 |
| Former smokers | 0.8 | 0.8 | -0.0010 |
| BMI >30 kg/m^2^ | 19.0 | 18.8 | 0.0050 |
| **Clinical history of CVD** |  |  |  |
| Peripheral artery disease | 2.3 | 2.4 | -0.0019 |
| Prior stroke | 4.3 | 4.3 | 0.0019 |
| **Outcomes** |  |  | ***P* value** |
| Premature ACS (≤67 years) presentation | 49.9 | 43.2 | <0.001 |
| Risk Ratio (95% CI) | 1.31 (1.25 – 1.38) | |  |
| Data are presented as percentages (%) or mean ± standard deviation, unless otherwise specified.  **Abbreviations:** ACS, acute coronary syndrome; BMI, body mass index; CAD, coronary artery disease; CVD, cardiovascular disease; PAD, peripheral artery disease. | | | |

| **Table S4.** Clinical factors and premature incidence of acute coronary syndrome stratified by diabetes status in women: Inverse probability weighting | | | |
| --- | --- | --- | --- |
|  | **Women** | |  |
| **Characteristics** | **Diabetes**  **(n = 6,953)** | **No Diabetes**  **(n = 18,537)** | **Standardized difference** |
| **Coronary heart disease risk factors** | | | |
| Hypercholesterolemia | 43.8 | 43.6 | 0.0046 |
| Hypertension | 69.3 | 69.3 | -0.0001 |
| Current Smokers | 25.8 | 25.9 | -0.0034 |
| Family history of CAD | 29.6 | 30.0 | -0.0093 |
| Former smokers | 0.8 | 0.8 | -0.0020 |
| BMI >30 kg/m^2^ | 18.5 | 18.7 | -0.0063 |
| **Clinical history of CVD** |  |  |  |
| PAD | 2.3 | 2.3 | -0.0002 |
| Prior stroke | 4.3 | 4.2 | 0.0045 |
| **Outcomes** |  |  | ***P* value** |
| Premature ACS (≤67 years) presentation | 39.9 | 47.6 | <0.001 |
| Risk Ratio (95% CI) | 0.73 (0.69 – 0.77) | |  |
| Data are presented as percentages (%) or mean ± standard deviation, unless otherwise specified.  **Abbreviations:** ACS, acute coronary syndrome; BMI, body mass index; CAD, coronary artery disease; CVD, cardiovascular disease; PAD, peripheral artery disease. | | | |

| **Table S5.** Clinical factors and premature incidence of acute coronary syndrome stratified by hypertension status in women: Inverse probability weighting | | | |
| --- | --- | --- | --- |
|  | **Women** | |  |
| **Characteristics** | **Hypertension**  **(n = 17,665)** | **No Hypertension**  **(n = 7,825)** | **Standardized difference** |
| **Coronary heart disease risk factors** | | | |
| Hypercholesterolemia | 43.5 | 43.5 | 0.0005 |
| Diabetes | 27.3 | 27.1 | 0.0049 |
| Current Smokers | 26.3 | 27.6 | -0.0282 |
| Family history of CAD | 30.0 | 29.6 | 0.0101 |
| Former smokers | 0.8 | 0.7 | 0.0144 |
| BMI >30, kg/m^2^ | 18.7 | 18.1 | 0.0153 |
| **Clinical history of CVD** |  |  |  |
| PAD | 2.3 | 2.3 | 0.0017 |
| Prior stroke | 4.2 | 4.1 | 0.0059 |
| **Outcomes** |  |  | ***P* value** |
| Premature ACS (≤67 years) presentation | 43.0 | 52.6 | <0.001 |
| Risk Ratio (95% CI) | 0.68 (0.65 – 0.72) | |  |
| Data are presented as percentages (%) or mean ± standard deviation, unless otherwise specified.  **Abbreviations:** ACS, acute coronary syndrome; BMI, body mass index; CAD, coronary artery disease; CVD, cardiovascular disease; PAD, peripheral artery disease. | | | |

| **Table S6.** Clinical factors and premature incidence of acute coronary syndrome stratified by smoking status in men: Inverse probability weighting | | | |
| --- | --- | --- | --- |
|  | **Men** | |  |
| **Characteristics** | **Current Smokers (n = 21,227)** | **Non**  **Current Smokers**  **(n = 24,229)** | **Standardized difference** |
| **Coronary heart disease risk factors** | | | |
| Diabetes | 19.9 | 20.0 | -0.0033 |
| Hypertension | 59.4 | 59.8 | -0.0080 |
| Hypercholesterolemia | 40.7 | 40.9 | -0.0048 |
| Family history of CAD | 29.4 | 29.4 | -0.0008 |
| Former smokers | 0.0 | 0.3 | -0.0374 |
| BMI >30 kg/m^2^ | 20.2 | 20.4 | -0.0050 |
| **Clinical history of CVD** |  |  |  |
| PAD | 2.3 | 2.4 | -0.0035 |
| Prior stroke | 3.7 | 3.7 | 0.0015 |
| **Outcomes** |  |  | ***P* value** |
| Premature ACS (≤63years) presentation | 68.8 | 43.9 | <0.001 |
| Risk Ratio (95% CI) | 2.82 (2.71 – 2.93) | |  |
| Data are presented as percentages (%) or mean ± standard deviation, unless otherwise specified.  **Abbreviations:** ACS, acute coronary syndrome; BMI, body mass index; CAD, coronary artery disease; CVD, cardiovascular disease; PAD, peripheral artery disease. | | | |

| **Table S7.** Clinical factors and premature incidence of acute coronary syndrome stratified by hypercholesterolemia status in men: Inverse probability weighting | | | |
| --- | --- | --- | --- |
|  | **Men** | |  |
| **Characteristics** | **Hypercholesterolemia (n = 18,575)** | **No**  **Hypercholesterolemia**  **(n = 26,888)** | **Standardized difference** |
| **Coronary heart disease risk factors** | | | |
| Diabetes | 20.0 | 19.8 | 0.0050 |
| Hypertension | 60.0 | 59.5 | 0.0093 |
| Current Smokers | 48.0 | 47.1 | 0.0170 |
| Family history of CAD | 29.6 | 29.4 | 0.0045 |
| Former smokers | 2.3 | 2.3 | 0.0011 |
| BMI >30 kg/m^2^ | 20.5 | 20.5 | 0.0011 |
| **Clinical history of CVD** |  |  |  |
| Peripheral artery disease | 2.4 | 2.3 | 0.0025 |
| Prior stroke | 3.7 | 3.7 | 0.0030 |
| **Outcomes** |  |  | ***P* value** |
| Premature ACS (≤63 years) presentation | 60.5 | 52.4 | <0.001 |
| Risk Ratio (95% CI) | 1.39 (1.34 – 1.45) | |  |
| Data are presented as percentages (%) or mean ± standard deviation, unless otherwise specified.  **Abbreviations:** ACS, acute coronary syndrome; BMI, body mass index; CAD, coronary artery disease; CVD, cardiovascular disease; PAD, peripheral artery disease. | | | |

| **Table S8.** Clinical factors and premature incidence of acute coronary syndrome stratified by diabetes status in men: Inverse probability weighting | | | |
| --- | --- | --- | --- |
|  | **Men** | |  |
| **Characteristics** | **Diabetes**  **(n = 9,035)** | **No Diabetes**  **(n = 36,428)** | **Standardized difference** |
| **Coronary heart disease risk factors** | | | |
| Hypercholesterolemia | 40.7 | 40.8 | -0.0040 |
| Hypertension | 59.2 | 59.4 | -0.0047 |
| Current Smokers | 47.3 | 46.7 | 0.0108 |
| Family history of CAD | 29.1 | 29.3 | -0.0052 |
| Former smokers | 2.3 | 2.3 | -0.0017 |
| BMI >30 kg/m^2^ | 20.2 | 20.3 | -0.0035 |
| **Clinical history of CVD** |  |  |  |
| Peripheral artery disease | 2.4 | 2.4 | 0.0027 |
| Prior stroke | 3.8 | 3.7 | 0.0034 |
| **Outcomes** |  |  | ***P* value** |
| Premature ACS (≤63 years) presentation | 49.1 | 57.1 | <0.001 |
| Risk Ratio (95% CI) | 0.73 (0.69 – 0.76) | |  |
| Data are presented as percentages (%) or mean ± standard deviation, unless otherwise specified.  **Abbreviations:** ACS, acute coronary syndrome; BMI, body mass index; CAD, coronary artery disease; CVD, cardiovascular disease; PAD, peripheral artery disease. | | | |

| **Table S9.** Clinical factors and premature incidence of acute coronary syndrome stratified by hypertension status in men: Inverse probability weighting | | | |
| --- | --- | --- | --- |
|  | **Men** | |  |
| **Characteristics** | **Hypertension**  **(n = 27,013)** | **No Hypertension**  **(n = 18,450)** | **Standardized difference** |
| **Coronary heart disease risk factors** | | | |
| Hypercholesterolemia | 40.8 | 40.7 | 0.0027 |
| Diabetes | 19.9 | 19.7 | 0.0034 |
| Current Smokers | 47.1 | 47.5 | -0.0075 |
| Family history of CAD | 29.4 | 29.3 | 0.0020 |
| Former smokers | 2.3 | 2.3 | 0.0027 |
| BMI >30 kg/m^2^ | 20.3 | 20.1 | 0.0057 |
| **Clinical history of CVD** |  |  |  |
| PAD | 2.4 | 2.3 | 0.0023 |
| Prior stroke | 3.7 | 3.6 | 0.0035 |
| **Outcomes** |  |  | ***P* value** |
| Premature ACS (≤63 years) presentation | 51.0 | 62.3 | <0.001 |
| Risk Ratio (95% CI) | 0.63 (0.61 – 0.65) | |  |
| Data are presented as percentages (%) or mean ± standard deviation, unless otherwise specified.  **Abbreviations:** ACS, acute coronary syndrome; BMI, body mass index; CAD, coronary artery disease; CVD, cardiovascular disease; PAD, peripheral artery disease. | | | |

| **Table S10.** Clinical factors and death from premature acute coronary syndrome stratified by smoking status in women: Inverse probability weighting | | | |
| --- | --- | --- | --- |
|  | **Women** | |  |
| **Characteristics** | **Current Smokers**  **(n = 6,626)** | **Non**  **Current Smokers**  **(n = 18,864)** | **Standardized difference** |
| **Coronary heart disease risk factors** |  |  |  |
| Diabetes | 27.8 | 27.1 | 0.0169 |
| Hypertension | 69.4 | 69.2 | 0.0038 |
| Hypercholesterolemia | 43.2 | 43.5 | -0.0061 |
| Family history of CAD | 30.1 | 30.3 | -0.0040 |
| Former smokers | 0.0 | 0.8 | -0.0861 |
| BMI >30 kg/m^2^ | 18.9 | 18.8 | 0.0023 |
| **Clinical history of CVD** |  |  |  |
| PAD | 2.2 | 2.3 | -0.0087 |
| Prior stroke | 4.1 | 4.2 | -0.0044 |
| **Outcome** |  |  | ***P* value** |
| Death from premature ACS (≤67years) presentation | 2.2 | 2.7 | 0.03 |
| Risk Ratio (95% CI) | 0.82 (0.68 – 0.98) | |  |
| Data are presented as percentages (%) or mean ± standard deviation, unless otherwise specified.  **Abbreviations:** ACS, acute coronary syndrome; BMI, body mass index; CAD, coronary artery disease; CVD, cardiovascular disease; PAD, peripheral artery disease. | | | |

| **Table S11.** Clinical factors and death from premature acute coronary syndrome stratified by hypercholesterolemia status in women: Inverse probability weighting | | | |
| --- | --- | --- | --- |
|  | **Women** | |  |
| **Characteristics** | **Hypercholesterolemia (n = 11,083)** | **No**  **Hypercholesterolemia**  **(n = 14,407)** | **Standardized difference** |
| **Coronary heart disease risk factors** | | | |
| Diabetes | 27.8 | 27.3 | 0.0107 |
| Hypertension | 70.4 | 69.5 | 0.0181 |
| Current Smokers | 27.2 | 26.6 | 0.0136 |
| Family history of CAD | 30.4 | 30.3 | 0.0038 |
| Former smokers | 0.8 | 0.8 | -0.0010 |
| BMI>30 kg/m^2^ | 19.0 | 18.8 | 0.0050 |
| **Clinical history of CVD** |  |  |  |
| PAD | 2.3 | 2.4 | -0.0019 |
| Prior stroke | 4.3 | 4.3 | 0.0019 |
| **Outcomes** |  |  | ***P* value** |
| Death from premature ACS (≤67years) presentation | 2.2 | 2.7 | 0.01 |
| Risk Ratio (95% CI) | 0.81 (0.69 – 0.96) | |  |
| Data are presented as percentages (%) or mean ± standard deviation, unless otherwise specified.  **Abbreviations:** ACS, acute coronary syndrome; BMI, body mass index; CAD, coronary artery disease; CVD, cardiovascular disease; PAD, peripheral artery disease. | | | |

| **Table S12.** Clinical factors and death from premature acute coronary syndrome stratified by diabetes status in women: Inverse probability weighting | | | |
| --- | --- | --- | --- |
|  | **Women** | |  |
| **Characteristics** | **Diabetes**  **(n = 6,953)** | **No** **Diabetes**  **(n = 18,537)** | **Standardized difference** |
| **Coronary heart disease risk factors** | | | |
| Hypercholesterolemia | 43.8 | 43.6 | 0.0046 |
| Hypertension | 69.3 | 69.3 | -0.0001 |
| Current Smokers | 25.8 | 25.9 | -0.0034 |
| Family history of CAD | 29.6 | 30.0 | -0.0093 |
| Former smokers | 0.8 | 0.8 | -0.0020 |
| BMI >30 kg/m^2^ | 18.5 | 18.7 | -0.0063 |
| **Clinical history of CVD** |  |  |  |
| PAD | 2.3 | 2.3 | -0.0002 |
| Prior stroke | 4.3 | 4.2 | 0.0045 |
| **Outcomes** |  |  | ***P* value** |
| Death from premature ACS (≤67years) presentation | 3.3 | 2.2 | <0.001 |
| Risk Ratio (95% CI) | 1.52 (1.29 – 1.79) | |  |
| Data are presented as percentages (%) or mean ± standard deviation, unless otherwise specified.  **Abbreviations:** ACS, acute coronary syndrome; BMI, body mass index; CAD, coronary artery disease; CVD, cardiovascular disease; PAD, peripheral artery disease. | | | |

| **Table S13.** Clinical factors and death from premature acute coronary syndrome stratified by hypertension status in women: Inverse probability weighting | | | |
| --- | --- | --- | --- |
|  | **Women** | |  |
| **Characteristics** | **Hypertension**  **(n = 17,665)** | **No Hypertension**  **(n = 7,825)** | **Standardized difference** |
| **Coronary heart disease risk factors** | | | |
| Hypercholesterolemia | 43.5 | 43.5 | 0.0005 |
| Diabetes | 27.3 | 27.1 | 0.0049 |
| Current Smokers | 26.3 | 27.6 | -0.0282 |
| Family history of CAD | 30.0 | 29.6 | 0.0101 |
| Former smokers | 0.8 | 0.7 | 0.0144 |
| BMI >30 kg/m^2^ | 18.7 | 18.1 | 0.0153 |
| **Clinical history of CVD** |  |  |  |
| PAD | 2.3 | 2.3 | 0.0017 |
| Prior stroke | 4.2 | 4.1 | 0.0059 |
| **Outcomes** |  |  | ***P* value** |
| Death from premature ACS (≤67years) presentation | 2.0 | 3.5 | <0.001 |
| Risk Ratio (95% CI) | 0.56 (0.48 – 0.66) | |  |
| Data are presented as percentages (%) or mean ± standard deviation, unless otherwise specified.  **Abbreviations:** ACS, acute coronary syndrome; BMI, body mass index; CAD, coronary artery disease; CVD, cardiovascular disease; PAD, peripheral artery disease. | | | |

| **Table S14.** Clinical factors and death from premature acute coronary syndrome stratified by smoking status in men: Inverse probability weighting | | | |
| --- | --- | --- | --- |
|  | **Men** | |  |
| **Characteristics** | **Current Smokers**  **(n = 21,227)** | **Non**  **Current Smokers**  **(n = 24,229)** | **Standardized difference** |
| **Coronary heart disease risk factors** |  |  |  |
| Diabetes | 19.9 | 20.0 | -0.0033 |
| Hypertension | 59.4 | 59.8 | -0.0080 |
| Hypercholesterolemia | 40.7 | 40.9 | -0.0048 |
| Family history of CAD | 29.4 | 29.4 | -0.0008 |
| Former smokers | 0.0 | 0.3 | -0.0374 |
| BMI >30 kg/m^2^ | 20.2 | 20.4 | -0.0050 |
| **Clinical history of CVD** |  |  |  |
| PAD | 2.3 | 2.4 | -0.0035 |
| Prior stroke | 3.7 | 3.7 | 0.0015 |
| Death from premature ACS (≤63years) presentation | 2.0 | 2.1 | 0.67 |
| Risk Ratio (95% CI) | 0.97 (0.85 – 1.11) | |  |
| Data are presented as percentages (%) or mean ± standard deviation, unless otherwise specified.  **Abbreviations:** ACS, acute coronary syndrome; BMI, body mass index; CAD, coronary artery disease; CVD, cardiovascular disease; PAD, peripheral artery disease. | | | |

| **Table S15.** Clinical factors and death from premature acute coronary syndrome stratified by hypercholesterolemia status in men: Inverse probability weighting | | | |
| --- | --- | --- | --- |
|  | **Men** | |  |
| **Characteristics** | **Hypercholesterolemia (n = 18,575)** | **No**  **Hypercholesterolemia**  **(n = 26,888)** | **Standardized difference** |
| **Coronary heart disease risk factors** | | | |
| Diabetes | 20.0 | 19.8 | 0.0050 |
| Hypertension | 60.0 | 59.5 | 0.0093 |
| Current Smokers | 48.0 | 47.1 | 0.0170 |
| Family history of CAD | 29.6 | 29.4 | 0.0045 |
| Former smokers | 2.3 | 2.3 | 0.0011 |
| BMI >30 kg/m^2^ | 20.5 | 20.5 | 0.0011 |
| **Clinical history of CVD** |  |  |  |
| PAD | 2.4 | 2.3 | 0.0025 |
| Prior stroke | 3.7 | 3.7 | 0.0030 |
| **Outcomes** |  |  | ***P* value** |
| Death from premature ACS (≤63years) presentation | 1.6 | 2.2 | <0.001 |
| Risk Ratio (95% CI) | 0.72 (0.63 – 0.83) | |  |
| Data are presented as percentages (%) or mean ± standard deviation, unless otherwise specified.  **Abbreviations:** ACS, acute coronary syndrome; BMI, body mass index; CAD, coronary artery disease; CVD, cardiovascular disease; PAD, peripheral artery disease. | | | |

| **Table S16.** Clinical factors and death from premature acute coronary syndrome stratified by diabetes status in men: Inverse probability weighting | | | |
| --- | --- | --- | --- |
|  | **Men** | |  |
| **Characteristics** | **Diabetes**  **(n = 9,035)** | **No Diabetes**  **(n = 36,428)** | **Standardized difference** |
| **Coronary heart disease risk factors** | | | |
| Hypercholesterolemia | 40.7 | 40.8 | -0.0040 |
| Hypertension | 59.2 | 59.4 | -0.0047 |
| Current Smokers | 47.3 | 46.7 | 0.0108 |
| Family history of CAD | 29.1 | 29.3 | -0.0052 |
| Former smokers | 2.3 | 2.3 | -0.0017 |
| BMI >30 kg/m^2^ | 20.2 | 20.3 | -0.0035 |
| **Clinical history of CVD** |  |  |  |
| PAD | 2.4 | 2.4 | 0.0027 |
| Prior stroke | 3.8 | 3.7 | 0.0034 |
| **Outcomes** |  |  | ***P* value** |
| Death from premature ACS (≤63years) presentation | 2.9 | 1.8 | <0.001 |
| Risk Ratio (95% CI) | 1.63 (1.41 – 1.89) | |  |
| Data are presented as percentages (%) or mean ± standard deviation, unless otherwise specified.  **Abbreviations:** ACS, acute coronary syndrome; BMI, body mass index; CAD, coronary artery disease; CVD, cardiovascular disease; PAD, peripheral artery disease. | | | |

| **Table S17.** Clinical factors and death from premature acute coronary syndrome stratified by hypertension status in men: Inverse probability weighting | | | |
| --- | --- | --- | --- |
|  | **Men** | |  |
| **Characteristics** | **Hypertension**  **(n = 27,013)** | **No Hypertension**  **(n = 18,450)** | **Standardized difference** |
| **Coronary heart disease risk factors** | | | |
| Hypercholesterolemia | 40.8 | 10.7 | 0.0027 |
| Diabetes | 19.9 | 19.7 | 0.0034 |
| Current Smokers | 47.1 | 47.5 | -0.0075 |
| Family history of CAD | 29.3 | 29.3 | 0.0020 |
| Former smokers | 2.3 | 2.3 | 0.0027 |
| BMI≥30 kg/m^2^ | 20.3 | 20.1 | 0.0057 |
| **Clinical history of CVD** |  |  |  |
| PAD | 2.4 | 2.3 | 0.0023 |
| Prior stroke | 3.7 | 3.6 | 0.0035 |
| **Outcomes** |  |  | ***P* value** |
| Death from premature ACS (≤63years) presentation | 1.6 | 2.6 | <0.001 |
| Risk Ratio (95% CI) | 0.59 (0.52 – 0.68) | |  |
| Data are presented as percentages (%) or mean ±standard deviation, unless otherwise specified.  **Abbreviations:** ACS, acute coronary syndrome; BMI, body mass index; CAD, coronary artery disease; CVD, cardiovascular disease; PAD, peripheral artery disease. | | | |

| **Table S18.** Interaction test for comparing two estimated risk ratios (women vs men) : incidence of premature acute coronary syndrome with current smoking: | | | |
| --- | --- | --- | --- |
|  |  | **Group 1**  **[Women]**  **(n = 25,490)** | **Group 2**  **[Men]**  **(n = 45,456)** |
| **1** | **RR** | 3.96 | 2.82 |
| **2** | **log RR** | 1.38 | 1.04 |
| **3** | **95% CI for RR** | 3.72 – 4.20 | 2.71 – 2.93 |
| **4** | **95% CI for log RR** | 1.31-1.44 | 1.00-1.08 |
| **5** | **Width of CI** | 0.12 | 0.08 |
| **6** | **SE (=width / (2*1.96))** | 0.03 | 0.02 |
|  | | | |
| **7** | **d (=**$\boldsymbol{E}_{\boldsymbol{1}}\boldsymbol{-}\boldsymbol{E}_{\boldsymbol{2}}$**)** | **0.34** | |
| **8** | **SE (d)** | **0.07** | |
| **9** | **CI (d)** | 0.27-0.41 | |
| **10** | **Test of Interaction** | 9.22 (***P* value<0.001**) | |
|  | | | |
| **11** | **RRR (=exp(d) )** | 1.40 | |
| **12** | **CI (RRR)** | 1.31-1.51 | |

| **Table S19.** Interaction test for comparing two estimated risk ratios (women vs men): incidence of premature acute coronary syndrome with hypercholesterolemia: | | | |
| --- | --- | --- | --- |
|  |  | **Group 1**  **[Women]**  **(n = 25,490)** | **Group 2**  **[Men]**  **(n = 45,463)** |
| **1** | **RR** | 1.31 | 1.39 |
| **2** | **log RR** | 0.27 | 0.33 |
| **3** | **95% CI for RR** | (1.25 – 1.38) | (1.34-1.45) |
| **4** | **95% CI for log RR** | 0.22-0.32 | 0.29-0.37 |
| **5** | **Width of CI** | 0.10 | 0.08 |
| **6** | **SE (=width / (2*1.96))** | 0.03 | 0.02 |
|  | | | |
| **7** | **d (=**$\boldsymbol{E}_{\boldsymbol{1}}\boldsymbol{-}\boldsymbol{E}_{\boldsymbol{2}}$**)** | **0.06** | |
| **8** | **SE (d)** | **0.03** | |
| **9** | **CI (d)** | -0.12-0.003 | |
| **10** | **Test of Interaction** | -1.83 (***P* value 0.03**) | |
|  | | | |
| **11** | **RRR (=exp(d) )** | 1.81 | |
| **12** | **CI (RRR)** | 1.57 -2.11 | |

| **Table S20.** Interaction test for comparing two estimated risk ratios (women vs men): incidence of death from premature acute coronary syndrome with diabetes: | | | |
| --- | --- | --- | --- |
|  |  | **Group 1**  **[Women]**  **(n = 25,490)** | **Group 2**  **[Men]**  **(n = 45,463)** |
| **1** | **RR** | 1.52 | 1.63 |
| **2** | **log RR** | 0.42 | 0.49 |
| **3** | **95% CI for RR** | (1.29 – 1.79) | (1.41 – 1.89) |
| **4** | **95% CI for log RR** | 0.25-0.58 | -0.52 – (-0.35) |
| **5** | **Width of CI** | 0.33 | 0.29 |
| **6** | **SE (=width / (2*1.96))** | 0.08 | 0.07 |
|  | | | |
| **7** | **d (=**$\boldsymbol{E}_{\boldsymbol{1}}\boldsymbol{-}\boldsymbol{E}_{\boldsymbol{2}}$**)** | **0.07** | |
| **8** | **SE (d)** | **0.11** | |
| **9** | **CI (d)** | 0.29 – 0.15 | |
| **10** | **Test of Interaction** | -0.62 (***P* value=0.27**) | |
|  | | | |
| **11** | **RRR (=exp(d) )** | 0.93 | |
| **12** | **CI (RRR)** | 0.74-1.16 | |

# **REFERENCES**

1. Bugiardini R, Badimon L, Investigators I-T, Coordinators. The International Survey of Acute Coronary Syndromes in Transitional Countries (ISACS-TC): 2010-2015. Int J Cardiol 2016;217 Suppl:S1-6.
2. Vasic A, Vasiljevic Z, Mickovski-Katalina N, Mandic-Rajcevic S, Soldatovic I. Temporal Trends in Acute Coronary Syndrome Mortality in Serbia in 2005-2019: An Age-Period-Cohort Analysis Using Data from the Serbian Acute Coronary Syndrome Registry (RAACS). Int J Environ Res Public Health 2022;19.
3. van Buuren, S, Groothuis-Oudshoorn K. "mice: Multivariate imputation by chained equations in R." Journal of Statistical Software. 2011;45(3). doi:10.18637/jss.v045.i03
4. Austin PC, Stuart EA. Moving towards best practice when using inverse probability of treatment weighting (IPTW) using the propensity score to estimate causal treatment effects in observational studies. *Stat Med* 2015; 34(28): 3661-79.
5. Katz D, Baptista J, Azen SP, et al. Obtaining Confidence Intervals for the Risk Ratio in Cohort Studies. *Biometrics* 1978;34(3):469-74. doi: 10.2307/2530610
6. Dongsheng Y, Dalton JE. A unified approach to measuring the effect size between two groups using SAS®. SAS Global Forum. Vol. 335. 2012.
7. Altman DG, Bland JM. Interaction revisited: the difference between two estimates. *BMJ* 2003; 326(7382): 219.
8. Wilkins E, Wilson L, Wickramasinghe K, et al. European Cardiovascular Disease Statistics 2017. European Heart Network, Brussels. https://ehnheart.org/cvd-statistics/cvd-statistics-2017.html.
